# Development of a keyword library for capturing PRO-CTCAE-focused “symptom talk” in oncology conversations

**DOI:** 10.1093/jamiaopen/ooad009

**Published:** 2023-02-09

**Authors:** Brigitte N Durieux, Samuel R Zverev, Elise C Tarbi, Anne Kwok, Kate Sciacca, Kathryn I Pollak, James A Tulsky, Charlotta Lindvall

**Affiliations:** Department of Psychosocial Oncology and Palliative Care, Dana-Farber Cancer Institute, Boston, Massachusetts, USA; Department of Psychosocial Oncology and Palliative Care, Dana-Farber Cancer Institute, Boston, Massachusetts, USA; NYU School of Medicine, New York University, New York, New York, USA; Department of Psychosocial Oncology and Palliative Care, Dana-Farber Cancer Institute, Boston, Massachusetts, USA; Department of Nursing, University of Vermont, Burlington, Vermont, USA; Department of Psychosocial Oncology and Palliative Care, Dana-Farber Cancer Institute, Boston, Massachusetts, USA; Department of Psychosocial Oncology and Palliative Care, Dana-Farber Cancer Institute, Boston, Massachusetts, USA; Department of Palliative Medicine, Brigham and Women’s Hospital, Boston, Massachusetts, USA; Department of Population Health Sciences, Duke University School of Medicine, Duke University, Durham, North Carolina, USA; Cancer Prevention and Control Program, Duke Cancer Institute, Duke University, Durham, North Carolina, USA; Department of Psychosocial Oncology and Palliative Care, Dana-Farber Cancer Institute, Boston, Massachusetts, USA; Department of Medicine, Brigham and Women’s Hospital, Boston, Massachusetts, USA; Department of Psychosocial Oncology and Palliative Care, Dana-Farber Cancer Institute, Boston, Massachusetts, USA; Department of Medicine, Brigham and Women’s Hospital, Boston, Massachusetts, USA; Harvard Medical School, Harvard University, Boston, Massachusetts, USA

**Keywords:** natural language processing, patient-reported outcome measures, computing methodologies, signs and symptoms, symptom assessment

## Abstract

**Objectives:**

As computational methods for detecting symptoms can help us better attend to patient suffering, the objectives of this study were to develop and evaluate the performance of a natural language processing keyword library for detecting symptom talk, and to describe symptom communication within our dataset to generate insights for future model building.

**Materials and Methods:**

This was a secondary analysis of 121 transcribed outpatient oncology conversations from the Communication in Oncologist-Patient Encounters trial. Through an iterative process of identifying symptom expressions via inductive and deductive techniques, we generated a library of keywords relevant to the Patient-Reported Outcome version of the Common Terminology Criteria for Adverse Events (PRO-CTCAE) framework from 90 conversations, and tested the library on 31 additional transcripts. To contextualize symptom expressions and the nature of misclassifications, we qualitatively analyzed 450 mislabeled and properly labeled symptom-positive turns.

**Results:**

The final library, comprising 1320 terms, identified symptom talk among conversation turns with an F1 of 0.82 against a PRO-CTCAE-focused gold standard, and an F1 of 0.61 against a broad gold standard. Qualitative observations suggest that physical symptoms are more easily detected than psychological symptoms (eg, anxiety), and ambiguity persists throughout symptom communication.

**Discussion:**

This rudimentary keyword library captures most PRO-CTCAE-focused symptom talk, but the ambiguity of symptom speech limits the utility of rule-based methods alone, and limits to generalizability must be considered.

**Conclusion:**

Our findings highlight opportunities for more advanced computational models to detect symptom expressions from transcribed clinical conversations. Future improvements in speech-to-text could enable real-time detection at scale.

## BACKGROUND AND SIGNIFICANCE

*“Everything about being sick is written in our bodies first and sometimes written in notebooks later*.”

– *Anne Boyer, The Undying*^1^

Among seriously ill patients, symptom burden is often unrecognized^1–4^ by clinicians despite being a top priority for patients, caregivers, and clinicians themselves.^5^ Symptoms experienced by cancer patients, from both disease and therapies, are numerous, exhausting^6^^,^^7^ and encompass physical and psychological domains.^8^^,^^9^ They affect quality of life, survival, medical costs, and functional status, among other significant patient-centered outcomes throughout the disease course.^10–13^ Among the many barriers to accurate symptom acknowledgement,^14^ documentation,^15^^,^^16^ and management^17^ are how patients attempt to communicate their symptom experience to clinicians, who may remember or log only a fraction of those reported.^2^^,^^18^^,^^19^

Whether due to communication dynamics, bias, or where these converge, clinicians miss symptoms. First, clinicians and patients may speak about symptoms in different ways^20^; when patients and clinicians communicate about symptoms (which they may not^21^), patients’ symptom descriptions are sometimes ambiguously understood by clinicians,^22^ clinicians’ language can include jargon,^23^ and communication may be more physician than patient-centered^24^ (given power relationships and other systemic factors [eg, trust]). Clinicians may interpret reported symptoms as over-reporting or not credible.^22^^,^^25^ Stigmas, for example around mental health^26^ or incontinence,^27^ may decrease patient reports of these symptoms. Additionally, symptoms may be overlooked due to factors related to patient identity and symptom type. More symptoms are missed in marginalized groups based on gender^28^ or race,^29^ psychological symptoms may be dismissed as a “normal” part of cancer,^30^ and there may be important gaps in perceptions of symptom severity between patients and their clinicians.^31^ Ultimately, when clinicians miss symptoms, they may miss opportunities for tending to suffering and delivering vital interventions such as early integration of palliative care.^32^

To standardize and better assess patients’ symptoms, Patient-Reported Outcome (PRO) measures^33^ and the Patient-Reported Outcome version of the Common Terminology Criteria for Adverse Events (PRO-CTCAE) symptom framework^34^ have become more routinely used in clinical care. While PRO tools are promising,^35^ gaps in patient use and completion^36^ limit their utility, which suggests the value of complementary data sources and assessment methods for monitoring symptoms at scale.

Computational methods (ie, natural language processing [NLP], machine learning, etc.) present opportunities to glean symptom information from a variety of data sources in a rapid, low-burden manner, which may support efforts to detect symptoms and improve care.^37^ Keyword libraries, used alongside NLP, are dictionaries of language which can be used to detect constructs of interest. These tools can be used on their own or to supplement the development and validation of more sophisticated AI models. Currently, efforts to detect symptoms via computational methods are evolving. AI models are being built to summarize medical conversations,^38^ and extract symptoms from clinical notes^39^^,^^40^; yet, research remains limited, specific to few symptoms, and largely lacks representation of verbal patient language.

Symptom keyword libraries have so far been generated from patient-authored text^41^ and written clinical notes^40^; there is not yet a public library base grounded in patients’ verbal conversation data. A library of this sort could help researchers detect symptoms from the rich, encompassing data source of audio-recorded clinical encounters, which are increasingly available with the rise of Telehealth and offer a high-impact setting for computational tools to record and summarize symptoms both in-the-moment and longitudinally.^37^

In this study, we built a keyword library from conversations between patients and oncology clinicians. Constructing our library around the PRO-CTCAE symptom framework, we based our library on language extracted from serious illness conversation transcripts from a multisite clinical trial.^42^ We evaluated library performance and explored the language participants used to describe their symptoms to generate insights for future model building. Increasingly, computational tools are being developed to monitor clinical conversations in real-time and provide in-the-moment and summative feedback (eg, about nonverbal communication behaviors).^43^ Creating this symptom keyword library moves us closer to the development of computational tools which can detect real-time symptom discussion. This can facilitate better symptom screening^44^ to identify burdened patients in need of care.

## OBJECTIVES

This is a secondary analysis of transcribed outpatient oncology encounters between oncologists, patients, and their caregivers (the conversation triad^45^). The objectives of this study were to (1) develop and evaluate the performance of a rudimentary NLP keyword library for detecting symptom talk, and (2) qualitatively analyze misclassifications and properly classified turns containing symptoms, in order to describe symptom communication within our dataset and generate insights for future model building. As detection was our goal rather than identification, we prioritized collecting as many relevant symptom terms as possible.

## MATERIALS AND METHODS

### Parent study and participants

All data came from the Communication in Oncologist-Patient Encounters (COPE) study,^42^ a randomized clinical trial aiming to improve patient expression of emotional concerns via online educational modules teaching strategies for communicating emotions, developed by palliative care physicians, psychologists, a software developer, and patient input.^42^ The trial consisted of audio-recorded outpatient oncology conversations at 2 academic medical centers in the United States between November 2010 and September 2014.

Patient participants were included in the COPE trial if they spoke English, were receiving care for advanced malignancy, had computer/email access, and were experiencing emotional difficulty. Emotional difficulty was evaluated by responses to the Impact of Events Scale; patients were included if they had a mean score of 1.375 or greater, or responded 5 (“often”) to at least one item. Patients were excluded if they had a documented diagnosis of active psychosis, dementia, or could not use a computer.

### Sample

In total, we analyzed 121 oncology conversations from 73 patients, split into 3 samples. Our ultimate sample size was determined by the number of COPE conversations which had been transcribed and annotated at the time of analysis (*n* = 286). Conversations were in the process of being sequentially human-transcribed (ADA Transcription, Westampton Township, NJ, USA) and annotated in “batches”; one batch was purposively enriched for patients with self-reported minority race/ethnicity. We excluded conversations which lacked patient ID data (*n* = 2), and in selecting our analytic samples, omitted conversations which contained speakers seen in a prior sample (*n* = 163).

#### Development sample

A random sample of 47 conversations representing 30 patients was used to create the initial draft of the keyword library.

#### Validation sample

A random sample of 43 conversations representing 28 patients, excluding speakers represented in the prior sample, was used to test preliminary library performance and inform expansions and adjustments.

#### Test sample

A sample of 31 conversations representing 15 patients, also excluding speakers from prior samples, was selected purposively to enrich for patients with self-reported minority race/ethnicity. We did this as our first 2 random samples included mostly White speakers^46^; as time and resource constraints limited our transcription capacity, we preferred to represent more minority patients in our work despite the demographic dissimilarity to our prior samples.

### Measures

Participants in the COPE study completed baseline measures which included demographic self-report data; race, ethnicity, marriage status, education, household financial situation, and household income. Patients’ primary oncologists completed a survey reporting sex, age, religion, race, and ethnicity.

### Gold standards for symptom talk

#### Broad gold standard

As part of a larger ongoing study seeking to build NLP models for detecting priority domains in serious illness conversations, 5 coders were trained to annotate COPE transcripts on the level of the speaker turn (an uninterrupted span of speech) for “symptom talk,” defined as:

“All references to symptoms and side effects of…cancer and its treatment, whether potential or actual, [present or absent]… (and) statements related to symptom management.”

Prior to annotating conversations, coders (3 clinicians and 2 research assistants) were trained extensively (studying the codebook, annotating sample conversations, and discussing questions); inter-rater reliability was calculated on 14 triple-coded conversations (Kappa = 0.63).

Our codebook was developed based on the literature^47–49^ and updated during the annotation process through regular discussions among the study team (Supplementary Appendix SA). To capture the ambiguities present in natural communication, annotators labeled turns on a 0–3 scale,^50^ with 3 implying certain relevance to symptoms. *Study team consensus determined that an average score of 2 or higher indicated symptom content (the “broad gold standard”).*

#### PRO-CTCAE-focused gold standard

As preliminary qualitative observations revealed that a difference in symptom targets between the broad gold standard and the PRO-CTCAE-specific library may complicate performance assessment, investigators created a human-validated gold standard specific to the targets of the library. *Two investigators [BND, SZ] prepared a second gold standard, based on the broad gold standard, manually modified to correct for annotator errors among turns and clarify whether they were PRO-CTCAE-relevant.*

### Keyword library development

A library of relevant terms was compiled through an iterative process of identifying symptom words using both retrospective and prospective techniques.

#### Definition of symptoms

This library was based on the National Cancer Institute’s PRO-CTCAE framework.^34^ Though the full Common Terminology Criteria for Adverse Events (CTCAE) list may encompass a broader range of patient-experienced symptoms, the PRO-based framework focuses on 80 most clinically relevant symptoms and makes for a more feasible symptom detection. We included words mentioning PRO-CTCAE-defined symptoms and related utterances, as well as medications commonly used to treat relevant symptoms (identified using Micromedex; IBM, Armonk, NY, USA).

#### Initial library draft

From a development sample of 47 conversation transcripts, we extracted all turns containing symptoms (according to the *broad gold standard*). Two coders [BND, SZ] went through all words spoken in these turns, viewing usage contexts using ClinicalRegex,^51^ and determining words and phrases for inclusion (decision tree illustrated in Supplementary Figure S1). Disagreements were adjudicated via discussion with the larger study group. All words and phrases were discussed and sorted into PRO-CTCAE symptom categories (nonexclusive); following this, a priori additions were gathered from prior work on clinical notes^40^ and further brainstormed as supplemental additions.

#### Library validation

A preliminary test of the initial library was performed on a validation sample of 43 conversations to inform necessary adjustments to the vocabulary. An NLP script searched transcripts on the conversation turn level for initial symptom keywords; results were compared against the 2 gold standards. Misclassified turns were screened for missed symptom terms and important exclusion cases, and adjustments applied for a final version of the library.

#### Library testing

The final library was tested against a sample of 31 conversations, with performance calculated against both gold standards. Results against the *broad gold standard* were investigated qualitatively to contextualize performance and generate insights for future model building; this analysis also made use of data from preliminary testing of the validation sample against the same gold standard.

### Analyses

#### Library use

NLP was used to search the validation and test datasets for library terms, barring library-defined exclusions. No additional regular expression rules were created.

#### Performance testing

Characteristics were estimated against gold standards as follows:

Sensitivity = TP/(TP + FN)

Specificity = TN/(TN + FP)

Precision = TP/(TP + FP)

Accuracy = (TP + TN)/(TP + TN + FP + FN)

F1 score = (2TP)/(2TP + FP + FN)

where TP refers to true positives (library correctly identified symptoms); FP, false positives (library falsely identified symptoms); FN, false negatives (library failed to identify symptoms); and TN, true negatives (no symptoms detected nor present).

#### Qualitative analysis

To contextualize library performance,^52^ we used content analysis^53^^,^^54^ to qualitatively analyze a random sample of 300 misclassified (FP, FN) and 150 properly classified (TP) speaker turns from tests against the *broad gold standard*, examining participants’ use of symptom keywords. Though randomly selected, turns were filtered to ensure even proportions of the Validation and Test Datasets and a diversity of conversations and uniquely represented patients. Three investigators [BND, SRV, and ECT] individually created coding schemes from 180 randomly chosen speaker turns. Investigators met to review the sample and resolve schemes to generate a codebook (Supplementary Table S1), which was applied by investigators [BND and SRV] to 2 additional samples (180 turns and 90 turns). Investigators met to review agreement, resolve disagreements, and reach consensus about additional codes and emerging findings.^55^ The total sample was deemed to be of sufficient information power to answer the study question.^56^

## RESULTS

### Participant sample

Table 1 characterizes patient participants (*n* = 73), and Table 2, oncologist participants (*n* = 40). Overall, patients were primarily White (*n* = 70%), non-Hispanic (99%), and married (78%). Just over half were male (52%); most patients had completed some college or more (82%). Patients’ household financial situations ranged, though three-quarters (75%) had an annual household income of $35 000 or greater. Patients’ primary oncologists were mostly male (78%), White (85%), and had a mean age of 46.5 years (SD: 9.9).

**Table 1.** Patient demographic characteristics

	Development sample (*n* = 30)	Validation sample (*n* = 28)	Test sample (*n* = 15)	Total (*n* = 73)
	(*n*, %)	(*n*, %)	(*n*, %)	(*n*, %)
Gender				
Female	19 (63%)	10 (36%)	5 (33%)	34 (47%)
Male	11 (37%)	18 (64%)	9 (60%)	38 (52%)
Missing	0	0	1 (7%)	1 (1%)
Race				
American Indian or Alaska Native	1 (3%)	0	1 (7%)^a^	2 (3%)^a^
Asian	0	1 (4%)	0	1 (1%)
Native Hawaiian or Other	0	2 (1 other) (7%)	2 (2 other) (13%)	4 (3 other) (5%)
Black/African American	2 (7%)	2 (7%)	11 (73%)^a^	17 (23%)^a^
White	27 (90%)	23 (82%)	1 (7%)	51 (70%)
More than one race	0	0	1 (7%)^a^	1 (1%)^a^
Unknown or not reported	0	0	1 (7%)	1 (1%)
Hispanic				
Yes	0	0	1 (7%)	1 (1%)
No	30 (100%)	28 (100%)	14 (93%)	72 (99%)
Marital status				
Married	24 (80%)	26 (93%)	7 (47%)	57 (78%)
Divorced or separated	4 (13%)	1 (4%)	4 (27%)	9 (12%)
Widowed	1 (3%)	1 (4%)	1 (7%)	3 (4%)
Never married	1 (3%)	0	3 (20%)	4 (5%)
Highest level of education				
Some high school	1 (3%)	0	0	1 (1%)
Completed high school or GED	7 (23%)	3 (11%)	1 (7%)	11 (15%)
Some college	4 (13%)	10 (36%)	6 (40%)	20 (27%)
Completed college	9 (30%)	7 (25%)	4 (27%)	20 (27%)
Graduate school	9 (30%)	8 (29%)	3 (20%)	20 (27%)
Missing	0	0	1 (7%)	1 (1%)
Household financial situation				
After paying the bills, you still have enough money for special things	18 (60%)	13 (46%)	5 (33%)	36 (49%)
You have enough money to pay the bills, but little spare money to buy extra or special things	7 (23%)	8 (29%)	3 (20%)	18 (25%)
You have the money to pay the bills, but only because you have cut back	3 (10%)	3 (11%)	3 (20%)	9 (12%)
You have difficulty paying the bills	2 (7%)	2 (7%)	4 (27%)	8 (11%)
Missing	0	2 (7%)	0	2 (3%)
Household income				
Less than $35 000	6 (20%)	1 (4%)	8 (53%)	15 (21%)
$35 000 or more	23 (77%)	25 (89%)	7 (47%)	55 (75%)
Missing	1 (3%)	2 (7%)	0	3 (4%)

a One patient reported this alongside another response.

**Table 2.** Primary oncologist demographic characteristics

	Development sample (*n* = 21)^a^	Validation sample (*n* = 9)	Test sample (*n* = 10)	Total (*n* = 40)^a^
	(*n*, %)	(*n*, %)	(*n*, %)	(*n*, %)
Gender				
Female	3 (14%)	1 (11%)	4 (40%)	8 (20%)
Male	17 (81%)	8 (89%)	6 (60%)	31 (78%)
Missing	1 (5%)	0	0	1 (2%)
Mean age in years [SD]	46.7 [9.8]	46.9 [10.1]	45.6 [10.9]	46.5 [9.9]
Religion				
Christian	11 (52%)	6 (67%)	6 (60%)	23 (58%)
Jewish	6 (29%)	2 (22%)	2 (20%)	10 (25%)
Islamic/Muslim	2 (10%)	0	1 (10%)	2 (5%)
Buddhist/Hindu/Eastern	0	0	0	0
No affiliation	1 (5%)	1 (11%)	1 (10%)	3 (8%)
Other	0	0	0	0
Missing	1 (5%)	0	0	1 (2%)
Race				
American Indian or Alaska Native	0	0	0	0
Asian	3 (14%)	0	2 (20%)^b^	5 (13%)^b^
Native Hawaiian or Other	1 (1 other) (5%)	0	0	1 (2%)
Black/African American	0	0	0	0
White	16 (76%)	9 (100%)	9 (90%)^b^	34 (85%)^b^
More than one race	0	0	1 (10%)^b^	1 (2%)^b^
Missing	1 (5%)	0	0	1 (2%)
Hispanic				
Yes	1 (5%)	0	1 (10%)	2 (5%)
No	19 (90%)	9 (100%)	9 (90%)	37 (93%)
Missing	1 (5%)	0	0	1 (2%)

a Survey data missing for 1 oncologist in the development sample.

b One patient reported this alongside another response.

### Performance against gold standards

Library performance was assessed against a *broad gold standard* (all “symptom talk”) and a *PRO-CTCAE-focused gold standard* (“symptom talk” specific to the PRO-CTCAE framework). Performance metrics are shown in Tables 3 and 4 and library iterations are available in Supplementary Appendix SB.

**Table 3.** Validation and test sample performance against 2 gold standards

	Validation sample (preliminary testing)		Test sample (final library testing)	
	vs broad gold standard	vs PRO-CTCAE-focused gold standard	vs broad gold standard	vs PRO-CTCAE-focused gold standard
True positives (TP)	1018	1381	921	1045
False positives (FP)	558	175	315	191
True negatives (TN)	18 744	19 424	12 556	13 157
False negatives (FN)	905	245	882	281
Total	21 225	21 225	14 674	14 674
Sensitivity	0.53	0.85	0.51	0.79
TP/(TP+FN)				
Specificity	0.97	0.99	0.98	0.99
TN/(TN+FP)				
Precision	0.65	0.89	0.75	0.85
TP/(TP+FP)				
Accuracy	0.93	0.98	0.92	0.97
TP+TN/(TP+TN+FP+FN)				
F1 score	0.58	0.87	0.61	0.82
2×TP/(2×TP+FP+FN)				

**Table 4.** Test sample performance versus broad gold standard, stratified by self-reported race

Patient race (*N*, number of conversations)	Sensitivity	Specificity	Precision	Accuracy	F1 score
Other (2, 5)	0.52	0.98	0.80	0.93	0.63
Black/African American (10, 21)	0.50	0.97	0.71	0.92	0.59
White (1, 1)	0.42	0.97	0.60	0.91	0.49
More than one race (1, 3)	0.51	0.99	0.86	0.95	0.64
Missing (1, 1)	0.55	0.99	0.88	0.92	0.67

#### Preliminary performance (validation sample)

The initial library comprised 1171 terms. When compared to the *broad gold standard*, this library captured symptom-containing turns with an F1 score of 0.58; when compared to the *PRO-CTCAE-focused gold standard*, the library captured relevant turns with an F1 score of 0.87. Postevaluation adjustments to the library included the exclusion of “deep breath(s)” and the addition of 158 terms (including “breathless,” “fuzzy brain,” and “painkiller”); full alterations appear in Supplementary Appendix SB.

#### Final library performance (test sample)

The adjusted keyword library included 1320 terms. When compared to the *broad gold standard*, the library captured symptom-containing turns with an F1 score of 0.61. When compared to the *PRO-CTCAE-focused gold standard*, the library captured relevant turns with an F1 score of 0.82. When performance was stratified by race, accuracy was similar across the board.

### Qualitative evaluation of misclassified turns and true positives

**Figure 1.**
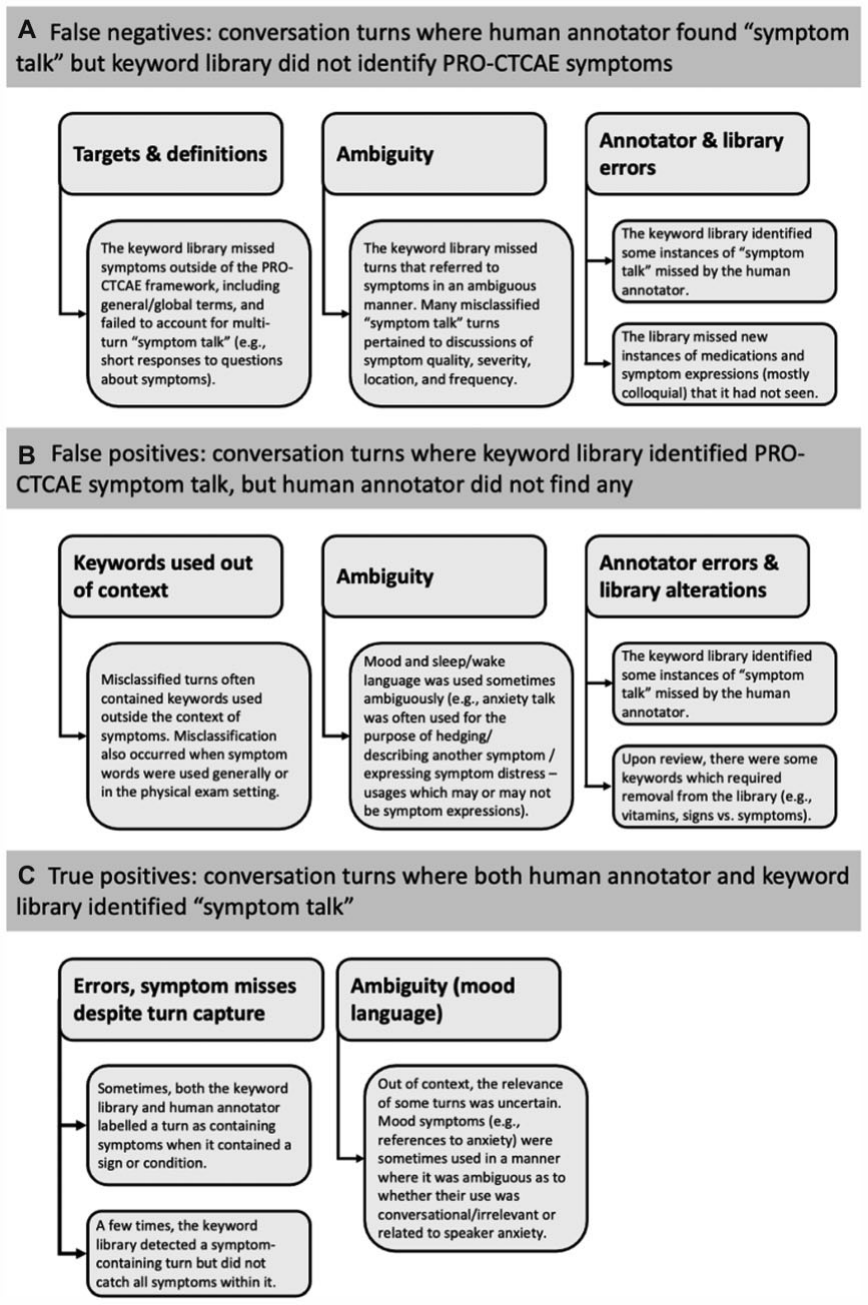
Observations among misclassified turns and true positives versus the broad gold standard. Misclassified turns and true positives from analysis against the human annotation labels for broad symptom talk revealed unique and overlapping insights. (A) Some false negatives did not appear to be library errors, often due to differences between targets, and in some cases, human annotator error. When the keyword library failed to catch symptom talk, error stemmed from ambiguous expressions or newly encountered language. (B) When the library falsely detected symptom talk, incorrect context or the ambiguous context of mood and anxiety language was often the basis for this error. The library sometimes detected turns with symptom expressions that had been missed by human annotators. (C) Among true positives, there remained some errors, often related to signs or conditions that might be associated with a symptom but are not symptoms themselves. There was an ambiguity to the context surrounding mood and anxiety expressions even among true positives.

In our analysis of 450 speaker turns representing false positives, false negatives, and true positives from library comparisons against the *broad gold standard*, 4 emergent insights contextualized library performance (Figure 1).

#### Conversational symptom discussion was often missed by a rule-based approach

The keyword library was designed to detect specific mention of PRO-CTCAE-defined symptoms. As a result, symptoms outside this framework were missed, as were those discussed conversationally (requiring multiple speaker turns to interpret). The latter often included general symptom discussion (eg, “how do you feel”), responses to previous turns containing symptoms or symptom-relevant questions (eg, “yes,” “no,” pain ratings such as “seven and three quarters”), and descriptions of symptom experience (eg, quality, severity, location, frequency, duration). The library also missed unique colloquial expressions (eg, “things will just run through” [diarrhea]), along with other newly encountered symptom terms or phrasings.

#### A keyword library may inaccurately detect symptoms, especially in cases of ambiguity and due to a lack of context

The keyword library identified moments in conversation where symptom language was used in a manner irrelevant to symptom experience. In some instances, keywords detected language used outside the context of symptoms (eg, “red”). In other cases, the context of a physical exam negated the term being a symptom description (eg, “deep breaths”). In some cases, language thought to indicate symptoms was instead used to introduce other topics (eg, “bothering you”). This was especially true with mood terms, which were often ambiguous; the same words used by patients to indicate anxiety were regularly used conversationally (eg, “don’t worry”).

In addition to speakers using symptom language outside of symptom talk, we observed speakers using condition-language when referring to symptom experience. For example, speakers used terms related to conditions as a reference point for health status (eg, cataracts, dementia). Keywords related to these instances were determined not to belong in the library.

#### The broad gold standard provided a useful comparison but may inflate error

Due to differing targets, persistent ambiguity, and in some cases, annotator error, only 67 of 150 false negative turns contained PRO-CTCAE-relevant symptom content (ie, less than half of these were indeed false negatives for the task the library was built for).

In about a third of the false positive turns, the library identified symptom-relevant turns where human annotators had missed symptom-relevant medications (eg, “stool softeners”), expressions containing symptom assessments and descriptions (eg, “painful”), or explicit symptom language (eg, “don’t have the appetite”).

#### Among true positives, almost all PRO-CTCAE symptom categories were represented; physical symptoms were more easily captured than psychological symptoms

Across the sample of 150 speaker turns, the most prevalent symptom categories mentioned were relevant to pain (*n* = 60) and GI (*n* = 34), followed by sleep/wake (*n* = 12), neurological (*n* = 11), and cutaneous (*n* = 11). Notably absent were mentions of oral or sexual symptoms.

Physical symptoms were more often mentioned in a clear manner than psychological symptoms. Some symptoms were assessed through general questions of medications (eg, “need any more Ativan” [anxiety medication]) or system well-being (eg, “how are your bowels”), but with these, physical symptoms tended to involve a smaller range of descriptions.

Mood and sleep/wake symptoms contained many instances of ambiguity, especially when observed in out-of-context speaker turns. Turns containing expressions of “worry” were identified both among true positives and among misclassified turns, suggesting human annotators had trouble classifying hedging statements using anxiety language (eg, “I’m worried about…”) and their relevance to symptoms.

## DISCUSSION

In this exploratory analysis of transcribed outpatient oncology encounters, we developed an NLP keyword library to capture symptom talk. We found that the library performed acceptably as a rudimentary tool for detecting PRO-CTCAE-defined symptoms (turn-level F1 score > 0.8, sensitivity ∼ 0.8) in our sample, though it is limited as a rule-based method. In qualitatively exploring misclassified turns and true positives, we observed that the library detected physical symptoms more easily than psychological symptoms, and that ambiguity persisted throughout symptom communication. Where some symptoms were communicated via consistent language, others were referred to by a range of colloquial expressions and described through quality descriptions, sometimes involving back-and-forth between speakers. Patients sometimes described symptoms using abstract language, underscoring challenges in using a keyword library to identify complex communication. Though we were not powered to analyze performance by patient race, group-level analyses necessitate further investigation.

Our findings align with previous work describing limitations of rule-based methods.^57–59^ Rule-based systems only capture predefined concepts, and are limited by the terms they search for; they often fail to generalize subtle variations in data (eg, misspellings), especially when it comes to accounting for the morphological variety of natural language.^57^ The back-and-forth nature of symptom communication^60^ may inherently effect our ability to infer if/what symptoms are being discussed, as is consistent with our observations of misclassifications. As they can account for context, more advanced computational tools such as pretrained language models^61^ may better perform the task of detecting symptoms, though limitations to generalizability remain with dataset shifts.^62^ Others have described that the limitations of rule-based methods are mitigated in cases where the tool acts in conjunction with a larger, stepwise process, as is often done in industry.^58^ Symptom detection via keywords can thus be useful as part of a larger information pipeline; for example, this library could be used in conjunction with human review using ClinicalRegex^51^^,^^63^ to identify conversations containing symptoms of interest.

Our qualitative findings examining misclassified turns and true positives are consistent with the literature: psychological and ambiguous symptom descriptions complicate symptom detection.^18–20^^,^^64^ In our study, symptoms were more easily captured if they were physical and described via common vernacular. In contrast, psychological symptoms such as anxiety were rarely discussed explicitly. Other researchers have referred to this difference as being between “objective symptoms” (eg, vomiting) and “subjective symptoms” (eg, fatigue)—they have highlighted that patient-report/clinician documentation concordance is higher for symptoms in the former category, which predicates symptom intervention.^18^ In this study, anxiety identification was particularly difficult, as “I’m worried” could indicate anxiety symptoms for one person and be innocuous for another. The study team experienced feeling more certain about anxiety medication talk being symptom-relevant as opposed to expressions of “worry.” Ultimately, anxiety exists on a spectrum and can be experienced below the threshold of a clinical disorder^65^; our findings align with literature describing the low sensitivity of mental disorder diagnosis outside the use of diagnostic tools.^66^

Our work has several limitations. Our library is extensive, but not exhaustive; keywords have been generated from study data and expanded on in an informed manner, but reflect the geographic and cultural language of the study participants and investigators and are therefore not generalizable alone. Notably, our sample size of both patients and clinicians was small, and disparate demographics among participants could lead to limited generalizability across all populations. Future investigators making use of this library should consider participant representation in including language specific to the use case and region, and should explore additional terms for inclusion. They should also consider whether they wish to omit symptom-specific or clinician-authored language in the library (indicated separately in Supplementary Appendix SB), or whether they wish to generate additional symptom keywords by employing other language models to extract key terms and phrases from relevant text.^67–69^ Ideally, future work should seek patient feedback on symptom terms; adding terms from crowdsourcing or surveys may provide a more comprehensive view of the many ways patients refer to symptoms in conversation. Another limitation is that transcription style and quality may affect what this library is able to capture^70^: biases in transcription choices may be propagated. Furthermore, using only written data for symptom detection may obfuscate important nonverbal or nontranscribed vocal communication cues, such as the speed or pitch of expressions. Applications employing this NLP tool should seek to capture important symptom communication from multiple data sources. This keyword library is inherently limited by using a limited symptom framework; PRO measures have been critiqued for lacking reliability, sensitivity, and acceptability when it comes to specific conditions (eg, psoriasis).^71^ Lastly, our sampling method made use of our data available at the time; our sequential transcription process may have biased our dataset temporally and in our representation of COPE participants.

This work presents the development of and provides rudimentary language for an NLP library to detect “symptom talk.” As other investigators have called upon NLP researchers to concentrate on symptoms and make vocabularies openly available,^72^ we present our keywords and encourage other investigators to refine and expand the language we have drafted. Our exploratory work suggests that this rule-based tool may be useful as part of a larger pipeline^51^; future work may employ this library in conjunction with more advanced methods (ie, to provide simple NLP performance metrics for comparison with more advanced ML during training, inform rules within hybrid models, etc.). Our hope is that automating some level of symptom detection from natural conversations may help detect symptom burden sooner, contributing to targeted, earlier integration of palliative care.^40^ Using conversation data may help us assess and address symptoms that go undocumented given their “expected” nature in cancer.^30^^,^^73^ As patient-provider communication is often implicated as barring cancer pain relief,^74^ involving computational methods in symptom detection could help capture symptoms that patients may not have explicit language for.

## CONCLUSION

Using an NLP keyword library, we can detect symptoms in both medical and colloquial verbiage as expressed by patients, families, and clinicians. This library warrants further development in more diverse datasets and settings, and should not be used alone but rather integrated into stepwise processes or otherwise involve human review. In future applications, whether to include medications and psychological symptom language should be considered carefully, as these are areas where conceptual grounding may differ from real use patterns. As we build algorithms to recognize symptom talk from conversations, supplementing standardized clinical terms with patient-centered language enhances our ability to recognize symptom burden in cancer, which may work toward the mitigation of symptom-related suffering.

## Supplementary Material

ooad009_Supplementary_Data

## Data Availability

The keyword library developed in this study is presented in Supplementary Appendix SB.
