# Supplementary material for: Development of a keyword library for capturing PRO-CTCAE-focused “symptom talk” in oncology conversations
Source: JAMIA Open. 2023 Feb 9;6(1):ooad009. doi: 10.1093/jamiaopen/ooad009 (PMC9912707; doi:10.1093/jamiaopen/ooad009)
Supplement: ooad009_Supplementary_Data [file ooad009_supplementary_data.zip › SKL_Appendix_B_1_18_23.docx]

**APPENDIX B: Keyword Library Iterations**

Symptom Keyword Library 1.1

| **PRO-CTCAE SYMPTOM [GROUPING / DOMAIN]** | **KEYWORDS / KEY PHRASES** |
| --- | --- |
| 1. Dry mouth [Oral] | biotene, biotene's, chewing gum, drier, dry mouth, dry throat, mouth dry, mouth dryness, mouth feeling dry, mouth is dry, mouth is even drier, mouth rinses, mouth seems dry, mouth stays dry, mouth stays real dry, throat feeling dry, throat gets dry, throat gets real dry |
| 2. Difficulty swallowing [Oral] | choking, doesn't seem to go down, even drinking just even water is hard, food gets stuck, hard to get down, difficulty swallowing, dysphagia, dysphasia, esophagitis, hard time swallowing, hard to swallow, haven't been able to swallow, problem swallowing, problems swallowing, protonix, swallow, swallowing, trouble swallowing |
| 3. Mouth/throat sores [Oral] | mouth bright red, mouth is bright red, mouth is red, mouth or throat sores, mouth sore, mouth sores, mouth/throat sores, mucositis, sore throat, soreness in her mouth, soreness in his mouth, soreness in my mouth, soreness in the mouth, soreness in the throat, soreness in your mouth, stomatitis, throat pain |
| 4. Cracking at the corners of the mouth (cheilosis/cheilitis) [Oral] | cheilitis, cheilosis, cracking at the corners of the mouth |
| 5. Voice quality changes [Oral] | losing her voice, losing his voice, losing my voice, losing your voice, change in voice quality, changes in voice quality, hoarseness, voice, voice change, voice changes, voice changing, voice heaviness, voice is changing, voice quality changes |
| 6. Hoarseness [Oral] | a little horse, a little hoarse, hoarse, hoarse voice, hoarseness |
| 7. Taste changes [Gastrointestinal] | can't taste, craving, don't enjoy meals, drown everything in hot sauce, little taste, tastes like cardboard, tastes like nothing, tastes metallic, don't taste, dysgeusia, gysguesia, problems with taste, taste changes |
| 8. Decreased appetite [Gastrointestinal] | anorexia, appetite, appetite change, appetite is just not really tere, appetite is just not there, appetite is low, appetite is not there, appetite is poor, appetite loss, appetite low, appetite off, appetite poor, appetite's, appetite's just not there, appetite's low, appetite's not there, appetite's pretty low, change in appetite, decreased appetite, decreased oral intake, don't have much appetite, don't have much of an appetite, early satiety, food appealing, forcing things down, hardly eat, haven't had an appetite, having a problem with food being appealing, lack of appetite, lose the desire to drink, lose the desire to eat, loss of appetite, lost her appetite, lost his appetite, lost my appetite, lost the desire to drink, lost the desire to eat, lost your appetite, low appetite, lower appetite, no appetite, not eating, poor appetite, poor PO intake, trouble eating, try even a bite, try to force things, unable to eat, wasn't appealing |
| 9. Nausea [Gastrointestinal] | queasiness, queasy, abhr, abhr gel, anti-nausea, compazine, kaopectate, kytril, nausea gel, zofran, nausea, nauseated, nauseation, nauseous |
| 10. Vomiting [Gastrointestinal] | abhr, abhr gel, compazine, dry heaves, dry heaving, emesis, kytril, puke, puked, pukes, puking, threw up, throw up, throwing up, thrown up, throws up, vomit, vomited, vomiting, vomits, zofran |
| 11. Heartburn [Gastrointestinal] | acid, acid blockers, acid buildup, acid reflux, antacids, apple cider vinegar in water, burning, burning after eating, burning around the throat, dyspepsia, elevate the head, gaviscon, gaviscons, gerd, head of your bed up, heartburn, indigestion, kaopectate, magnesium, milk of magnesia, milk of magnesium, nexium, pepcid, protonix, rabeprazole, rabon, reflux, regurgitate, regurgitates, regurgitating, severe burning, stomach's talking to me, tagamet, wedge pillow, wedge pillows, zantac |
| 12. Gas [Gastrointestinal] | burp, burping, burps, pass gas, pass the gas, simethicone, flatulence, flatus, gas, gassy |
| 13. Bloating [Gastrointestinal] | bloating-ness, blows up like a balloon, bomb went off, filled up with air, how big my stomach was, sounds like a drum, sounds like a tom tom or a drum, stomach blows up, stomach got really big, stomach was all big, stomach was extended, stomach was really extended, stomach's all big, abdominal distension, abdominal distention, bloated, bloating, distended, distension, distention, fullness, stomach swelling, swollen abdomen |
| 14. Hiccups [Gastrointestinal] | hiccup, hiccups |
| 15. Constipation [Gastrointestinal] | activia, backed up, backed-up, benefiber, bowel obstruction, bowels moving, calcium, colace, constipa, constipated, constipates, constipating, constipation, docusate, enema, fiber, gatorade prep, gets things moving, hard as a rock, hard stool, hard stools, indigestion, lack of bowel movement, laxative, magnesium, makes them easier to pass, makes your stool soft, makes your stools soft, metamucil, milk of magnesia, milk of magnesium, miralax, obstipation, prune juice, senna, senokot, soften things, stool softener, stool softeners, stools are hard, stools are so hard, stop up the bowels, stop up your bowels, straining, to strain, trouble pooping, when you strain |
| 16. Diarrhea [Gastrointestinal] | diarrhea, diarrhea's, gatorade, get my bowels slowed back down, get my bowels slowed down, get your bowels slowed back down, get your bowels slowed down, imodium, imodiums, it was water, it's all watery, it's watery, kaopectate, lomotil, ned, pedialyte, rumbling in the stomach, stomach is very rumbly, stool's too loose, stool's watery, stools become too loose, the depends, the runs, the trots, water mixed with loose stool, watery rush, watery stool |
| 17. Abdominal Pain [Gastrointestinal] | abd cramps, abd discomfort, abd pain, abdomen, abdominal cramp, abdominal cramps, abdominal discomfort, abdominal pain, discomfort in her abdomen, discomfort in his abdomen, discomfort in my abdomen, discomfort in your abdomen, epigastric discomfort, epigastric pain, heating pad, left upper quadrant discomfort, left upper quadrant pain, LUQ discomfort, LUQ pain, pain in her stomach, pain in his stomach, pain in my stomach, pain in the stomach, pain in your stomach, right upper quadrant discomfort, right upper quadrant pain, RUQ discomfort, RUQ pain, stomach ache, stomach hurting, stomach hurts, stomach's hurting, stomach pains, stomachache, upset my stomach, upset stomach, upset your stomach, "when I get these pains, I know I got to shit" |
| 18. Fecal Incontinence [Gastrointestinal] | bathroom, bowel, bowels moving, can't poop, comes out without even knowing it, continence, digest, digestive, fecal incontinence, had an accident, had some accidents, have an accident, having any accidents, incontinence, incontinent, not having any continence, not having any kind of continence, panty-type things, several accidents, specialized panties, stool, stool incontinence, stool's, toilet, "when he has to go, he has to go", "when he has to go, literally he has to go", "when I have to go, I have to go", "when she has to go, she has to go" |
| 19. Shortness of Breath [Respiratory] | ability to breathe, breath, breath is deteriorating, breathe, breathe lightly, breathing, breaths, can't catch my breath, can't catch your breath, catching my breath, catching your breath, choking, DOE, dyspnea, dyspnea on exertion, dyspnea with exertion, dyspneic, exertion, exertional dyspnea, exertional shortness of breath, huffing, huffing and puffing, labored breathing, limiting my breathing, limiting your breathing, not have enough oxygen, out of breath, outta breath, pulmicort, run short of oxygen, short of breath, short of breath with exertional activity, short of breaths, shortness of breath, shortness of breath on exertion, shortness of breath with activity, shortness of breath with exertion, SOB, SOB with exertion, take my breath away, winded |
| 20. Cough [Respiratory] | [coughing], [coughs], cough, cough suppressant, cough syrup, coughed, coughing, coughing], coughs, junky in my chest, pertussis, protonix, robitussin, strangle, strangling, strangulating, vicks |
| 21. Wheezing [Respiratory] | chest makes noise, chest making noise, heard a whistling, junky in my chest, pulmicort, wheezing |
| 22. Swelling [Cardio/Circulatory] | anti-swelling, bursitis, celebrex, edema, feel like I'm walking on big, lymphedema, retaining a lot of fluid, shoes got a little tight, swell, swelled, swelling, swellness, swollen, toes are squished |
| 23. Heart Palpitations [Cardio/Circulatory] | heart palpitation, heart palpitations, irregular heartbeat, irregular heartbeats, palpitations, racing heart, rapid heart rate |
| 24. Rash [Cutaneous] | bunch of little bumps, blotches, cellulitis, dermatitis, eruption, face rash, lesions, marks, rash, rashes, reaction, red, red blotches, shingles, skin rash |
| 25. Skin Dryness [Cutaneous] | alligator skin, aveeno, dry, dry around the heels, dry skin, dryness, it's dry, leathery, lotion, moisturizer, peeling, skin cracking, skin dryness, skin is dry, skin is so dry, skin is so thin, skin is thin, skin thin, thin skin, thin-skin working hands, xerosis |
| 26. Acne [Cutaneous] | acne |
| 27. Hair Loss [Cutaneous] | a little thinner, alopecia, eyebrows, eyelashes, fall out, falling out, fuzz, hair, hair loss, hair thinning, hair's, losing her hair, losing his hair, losing my hair, losing your hair, lost all her lair, lost all his hair, lost all my hair, lost all your hair, lost hair, lost her hair, lost his hair, lost my hair, lost your hair, mustache, thinning |
| 28. Itching [Cutaneous] | benadryl, don't scratch, I scratch, irritates, irritating, irritation, itch, itches, itchiness, itching, itchy, pruritic, pruritis |
| 29. Hives [Cutaneous] | bumps, hives, urticaria |
| 30. Hand-foot Syndrome [Cutaneous] | hand-foot, hand-foot syndrome, hand/foot, hand/foot syndrome, PPE |
| 31. Nail Loss [Cutaneous] | brittle nails, nail loss |
| 32. Nail Ridging [Cutaneous] | nail ridging |
| 33. Nail Discoloration [Cutaneous] | nail changes, nail discoloration |
| 34. Sensitivity to Sunlight [Cutaneous] | sensitivity to sunlight, sunburn |
| 35. Bed/pressure Sores [Cutaneous] | bed sore, bed sores, bedsore, bedsores, pressure sore, pressure sores, sore, sores |
| 36. Radiation Skin Reaction [Cutaneous] | dry skin comes up shaped just like the incision, injury from radiation, radiation injury, radiation skin reaction, radiation skin reactions, radiation treatment affected tissue, radiation-injured |
| 37. Skin Darkening [Cutaneous] | cortisone, color change, erythema, flushing, funniest looking spot, hyperpigmentation, hyperpigmented, jaundiced, redness, skin darkening, skin darker, spot on your skin |
| 38. Stretch Marks [Cutaneous] | stretch marks |
| 39. Numbness & Tingling [Neurological] | 10 mg PO, are more numb, are number, burning, burning pain, carpal tunnel, compression sock, compression stocking, cymbalta, dysesthesia, dysesthesias, dysesthia, fingertips, needles and pins, nerve damage, nerve injury, nerve pain, neuralgia, neuropathic, neuropathic pain, neuropathy, numb, numbness, numbness & tingling, numbness and tingling, numbness or tingling, numbness/tingling, paresthesia, paresthesias, permanent sensation, pins and needles, PN, sciatica, tingling, tingling and numbness, tingly, tips of my fingers, tips of your fingers |
| 40. Dizziness [Neurological] | black out, blackout, collapsed, collapsing, dizziness, dizzy, faint, fainted, fainting, light headed, light-headed, lightheaded, lightheadedness, lose consciousness, loss of consciousness, pass out, passed out, passing out, room's spinning, spell where you collapsed, vertigo, wobbly, woozy |
| 41. Blurred Vision [Visual/Perceptual] | blurred, blurred vision, blurry vision, cataract, cataracts, diplopia, double-vision, keep seeing stuff, problem with her vision, problem with his vision, problem with my vision, problem with vision, problem with your vision, vision, vision gray out, vision grays out, visual disturbance, visual disturbances |
| 42. Flashing Lights [Visual/Perceptual] | flashing lights, lights flashing, see funny things, see lights, see lights or funny things |
| 43. Visual Floaters [Visual/Perceptual] | floaters, visual floaters |
| 44. Watery Eyes [Visual/Perceptual] | eyes are watery, eyes water, eyes watering, eyes watery, tear up, watering eyes, watery eyes |
| 45. Ringing in Ears [Visual/Perceptual] | ears ringing, ringing in ears, ringing in her ears, ringing in his ears, ringing in my ears, ringing in your ears, tinnitus |
| 46. Concentration [Attention/Memory] | ability to focus, alert, alertness, attention, chemo brain, cognitive, cognitive slowing, cognitively, cognitively slower, concentrate,concentration, confused, confusion, decreased concentration, difficulty concentrating, difficulty focusing, disoriented, distracted, focused and paying attention, goofiness, hard to concentrate, loopy, mental plasticity, mental stamina, mental thing, mental things, mixing up, more alert, out of it, paying attention, problems with understanding, problems with your memory or your thinking, process of thinking, trouble concentrating, trouble really concentrating, trouble with thinking |
| 47. Memory [Attention/Memory] | dementia, difficulty remembering, forgetful, forgetfulness, memory, memory loss, memory's, mushkabobble, problems with your memory or your thinking, short-term memories, short-term memory |
| 48. General Pain [Pain] | acetaminophen, ache, aches, aches/pains, achiness, aching, achy, aggravating, agitates, agitating, agitation, aleve, balled up, bone grinding, bothering me, buckling over, celebrex, chest discomfort, chest pain, chest pain and heaviness, chest pressure, choking, codeine, codeine's, codone, cortisone, CP, cutting, cymbalta, darvocet, dilaudid, discomfort, excruciating, fentanyl, generalized pain, gnawing, hurt, hurting, hurts, hydrocodone, ibuprofen, ibuprofen/motrin, LBP, methadone, morphine, motrin, naproxen, narcotic, narcotics, norco, oxy, oxy's, oxycodone, oxycodones, oxycontin, pain, pain-medicine, pain's, painful, pains, percocet, pressure, pressure's, sensitivity, sharp, sharpness, shooting, sore, soreness, spiking, stabbing, tender, tenderness, tylenol, uncomfortable, vicodin |
| 49. Headache [Pain] | excedrin, HA, head feels heavy, headache, headaches, heavy head, migraine, migraines |
| 50. Muscle Pain [Pain] | ache, aches, achiness, body aches, charley horse, charley horses, cramp, cramping, cramps, crampy, cymbalta, muscle ache, muscle aches, muscles are sore, muscle pain, muscle spasm, muscle spasms, muscle tension, magnesium, myalgia, myalgias, naproxen, quinine, spasm, spasms, tonic, vicks |
| 51. Joint Pain [Pain] | aleve, arthralgia, arthralgias, arthri, arthritic, arthritic pain, arthritis, bursitis, celebrex, corticosteroid, cortisone, gout, ibuprofen, ibuprofen/motrin, in the joint, joint discomfort, joint issue, joint issues, joint pain, joint pains, joints are hurting, joints hurt, knee pain, motrin, naproxen, painful joints, rheumatoid, tendinitis, tendonitis |
| 52. Insomnia [Sleep/Wake] | ambien, ativan, benadryl, can't sleep, cannot sleep, cat napping, couldn't sleep, didn't sleep very well, difficulty falling asleep, difficulty sleeping, difficulty staying asleep, difficulty with sleep, disrupted sleep, don't get sleepy, don't get tired, dyssomnia, falling asleep issue, insomnia, interruptions in her sleep, interruptions in his sleep, interruptions in my sleep, interruptions in your sleep, keep me awake, keeping her awake, keeping him awake, keeping me awake, keeping you awake, keeps me awake, lorazepam, melatonin, not been sleeping, not really sleeping, not sleeping, poor sleep, sleep, sleep deprivation, sleep disturbance, sleep disturbances, sleeping, sleeping pill, still awake at, tossing and turning, trouble sleeping, up all night, wake up in the middle of the night, waking up in the middle of the night |
| 53. Fatigue [Sleep/Wake] | all the juice is gone, decreased energy, doesn't have much stamina, don't have much stamina, don't have stamina, dopey, dozing off, draggy, drowsy, energy level, exertion, exhausted, fatigue, fatigued, groggy, has to rest more often, have to rest more often, help with staying awake, helps with staying awake, I don't last very long, knock me out, knock you out, knocks her out, knocks him out, knocks me out, knocks you out, lack of energy, less energies, less energy, less stamina, low energy, make her sleepy, make him sleepy, make me sleepy, make you sleepy, makes her sleepy, makes him sleepy, makes me sleepy, makes you sleepy, more sleepy, passing out, poop out, run out of juice, sedation's really high, sleep all day, sleepiness, sleeps all day, sleepy, tire, tire more easily, tired, tiredness, tiring, too tired, unconscious, wipes her out, wipes him out, wipes me out, wipes you out, wiping her out, wiping him out, wiping me out, wiping you out, worn down, zapped |
| 54. Anxious [Mood] | afraid, agitation, anxieties, anxiety, anxiety symptoms, anxious, ativan, buspirone, can't relax, cymbalta, get panicked, gets panicked, jumpy, lorazepam, make you relax, mirtazapine, nervousness, panic, panic attack, panic attacks, panic disorder, panic spell, restless, start shaking inside, worried, worries, worry, worrying, xanax, zoloft |
| 55. Discouraged [Mood] | discouraged, disheartening, hopeless, hopelessness, listless, mood disorder, mood disorders, overwhelmed |
| 56. Sad [Mood] | amitriptyline, anguish, anti-depressant, anti-depressants, antidepressant, antidepressants, celexa, citalopram, cries, crying, cymbalta, depressed, depressed mood, depressing, depression, depressive, depressive sxs, depressive symptoms, down in the dumps, dysphoria, dysphoric mood, feel down, feeling a little more down, feeling blue, feeling down, feeling kind of blue, feeling kind of low, feeling low, grief, grieving, hurting yourself, locked up in the crazy house, low mood, low/depressed, low/depressed mood, mental facility, mirtazapine, mood, mood disorder, mood disorders, mood has been low, mood instability, mood is down, mood is low, mood issues, mood symptoms, mood toxicity, mood's been down, negative frame of mind, not herself mentally, not himself mentally, not myself mentally, not yourself mentally, psychiatric evaluation, psychiatric problems, psychologic, remeron, sad, sadness, seroquel, suicidal, suicidal thoughts, tearful, tearfulness, teary, zoloft |
| 57. Irregular Periods/Vaginal Bleeding [Genitourinary] | bleeding, irregular period, irregular periods, postmenopausal bleeding, vaginal bleeding, vaginal spotting |
| 58. Missed Expected Menstrual Period [Genitourinary] | menopause, menses, missed expected menstrual period, missed expected period, missed her period, missed my period, missed period, missed your period |
| 59. Vaginal Discharge [Genitourinary] | bloody discharge, some discharge, vaginal discharge |
| 60. Vaginal Dryness [Genitourinary] | vaginal dryness |
| 61. Painful Urination [Genitourinary] | hurt to pee, hurts to pee, pain during urination, pain while urinating, painful urination |
| 62. Urinary Urgency [Genitourinary] | changes in your urinary habits, urinary urgency |
| 63. Urinary Frequency [Genitourinary] | bladder, changes in your urinary habits, emptying her bladder, emptying his bladder, emptying my bladder, emptying your bladder, frequent urination, loss of bladder control, nocturia, polyuria, urinary frequency, urinating more frequently |
| 64. Change in Usual Urine Color [Genitourinary] | change in usual urine color, change in your urine, changes in your urine, unusual urine color |
| 65. Urinary Incontinence [Genitourinary] | bathroom, catheter, catheterization, changes in your urinary habits, comes out without even knowing it, every time I cough, had an accident, had some accidents, have an accident, having any accidents, I feel like I'm peeing, incontinence, incontinent, leaking, not having any continence, not having any kind of continence, panty-type things, peeing all over the place, several accidents, specialized panties, trouble urinating, urinary incontinence, uropathy, "when he has to go, he has to go", "when he has to go, literally he has to go", "when I have to go, I have to go", "when she has to go, she has to go" |
| 66. Achieve and Maintain Erection [Sexual] | achieve and maintain erection, erectile dysfunction |
| 67. Ejaculation [Sexual] | ejaculation |
| 68. Decreased Libido [Sexual] | decreased libido, low libido, no sex drive |
| 69. Delayed Orgasm [Sexual] | delayed orgasm |
| 70. Unable to have Orgasm [Sexual] | unable to have orgasm |
| 71. Pain w/ Sexual Intercourse [Sexual] | dyspareunia, pain during intercourse, pain during sex, pain during sexual intercourse, pain with sexual intercourse |
| 72. Breast Swelling and Tenderness [Miscellaneous] | breast pain, breast swelling, breast swelling and tenderness, breast swelling or tenderness, breast tenderness, discomfort, discomfort at the lumpectomy bed, swelling, swollen |
| 73. Bruising [Miscellaneous] | black and blue, black-and-blue, blue-ish, bruise, bruises, bruising |
| 74. Chills [Miscellaneous] | arctic, can't get warm, cannot get warm, chill, chilled, chilling, chills, cold and chilled, cold sensation, couldn't get warm, feel cold all the time, freezing, hot and cold, hot cold, hot/cold, shakiness, shaking, shiver, shivering, shivering/shaking, takes a while to get warm, teeth are chattering, teeth chattering, teeth have been chattering |
| 75. Increased Sweating [Miscellaneous] | codeine, diaphoresis, diaphoretic, increased sweating, night sweats, pillow is all wet, pillow is soaked, pillow is wet, soaked, soaking, soaking wet, sweat, sweating, sweats |
| 76. Decreased Sweating [Miscellaneous] | decreased sweating, less sweating |
| 77. Hot Flashes [Miscellaneous] | hot flash, hot flashes, hotflashes, temperature deregulation, temperature dysregulation, warming of the body |
| 78. Nosebleed [Miscellaneous] | bloody nose, bloody noses, epistaxis, nosebleed, nosebleeds |
| 79. Pain and Swelling at Injection Site [Miscellaneous] | injected, injection site, pain and swelling at injection site |
| 80. Body Odor [Miscellaneous] | body odor |

Symptom Keyword Library Additions Post 1.1

| **PRO-CTCAE SYMPTOM** | **KEYWORDS / KEY PHRASES** |
| --- | --- |
| 1. Dry mouth [Oral] | can't get it wet, cracks, parched |
| 7. Taste changes [Gastrointestinal] | doesn't really taste, food just turned me off, hard eating, how does food taste, no desire, no taste, smaller meals |
| 8. Decreased appetite [Gastrointestinal] | don't feel hungry, eating good, get hungry, hard eating, haven’t been hungry, megace, no desire, not hungry, smaller meals, yarrow root |
| 9. Nausea [Gastrointestinal] | inflammation of your stomach, meclizine, prednisone |
| 10. Vomiting [Gastrointestinal] | keeping it down, meclizine, prednisone |
| 11. Heartburn [Gastrointestinal] | aciphex, esomeprazole, inflammation of your stomach, rolaids, sucralfate |
| 13. Bloating [Gastrointestinal] | inflammation of your stomach, protruded |
| 15. Constipation [Gastrointestinal] | bowels, calciums, lactulose, probiotics, stools, you have this blockage |
| 16. Diarrhea [Gastrointestinal] | bowels, diarrheas, on the loose side, probiotics, slow your bowels down, stools, yarrow root |
| 17. Abdominal Pain [Gastrointestinal] | a stitch, bottom falls out |
| 18. Fecal Incontinence [Gastrointestinal] | bottom falls out, bowels, diaper, diaper pad, dribble in your underwear, dribbling, get to the restroom on time, soiled it, stools |
| 19. Shortness of Breath [Respiratory] | breathless, oxygen, puffing |
| 20. Cough [Respiratory] | delsym |
| 21. Wheezing [Respiratory] | puffing |
| 22. Swelling [Cardio/Circulatory] | dex, dexamethasone, fluid collection, hydrocortisone, prednisone, puffing, puffing up, puffy, steroid, steroids, toradol |
| 23. Heart Palpitations [Cardio/Circulatory] | heart is starting to race, lidocaine |
| 24. Rash [Cutaneous] | hydrocortisone, metrocream |
| 25. Skin Dryness [Cutaneous] | aquaphor, breakdown of your skin, eucerin, keep it moisturized, lubiderm, lubriderm, scaley, skin is more thin, vaseline |
| 26. Acne [Cutaneous] | metrocream |
| 27. Hair Loss [Cutaneous] | wig |
| 28. Itching [Cutaneous] | hydrocortisone, irritated |
| 29. Hives [Cutaneous] | blisters, metrocream |
| 31. Nail Loss [Cutaneous] | fingernails |
| 32. Nail Ridging [Cutaneous] | fingernails, like rings, ridges, the rings |
| 33. Nail Discoloration [Cutaneous] | become darker sometimes, fingernails, gonna be darker, like rings, the darkness, the rings |
| 34. Sensitivity to Sunlight [Cutaneous] | eucerin |
| 35. Bed/pressure Sores [Cutaneous] | blisters |
| 36. Radiation Skin Reaction [Cutaneous] | skin toxicity |
| 37. Skin Darkening [Cutaneous] | become darker sometimes, rosy cheeks, gonna be darker, the darkness |
| 39. Numbness & Tingling [Neurological] | bengay, compression damage, compression sleeve, didn't feel it, feet and legs, gauntlet, in the fingers, in the sole of the feet, just like buzz, less sensation, lidocaine, longer gauntlet, lose feeling, lyrica, nerves are doing a little better, nucynta, recover my feelings, stocking glove |
| 40. Dizziness [Neurological] | knocked her out, knocked him out, knocked me out, knocked you out, meclizine |
| 46. Concentration [Attention/Memory] | fog, fuzzy brain |
| 48. General Pain [Pain] | advil, aspirin, bengay, bothering you, breakthrough, chest heaviness, dex, dexamethasone, drives me bananas, epidurals, feel really bad, hydromorphone, it burns, kind of shoot, like arrows, lyrica, nucynta, numbing medication, numbing medicine, painkiller, prednisone, radiating up, steroid, steroids, toradol, tramadol, twinge, twinges |
| 49. Headache [Pain] | advil, aspirin |
| 50. Muscle Pain [Pain] | a stitch, bengay, diazepam, twinge, twinges, valium |
| 51. Joint Pain [Pain] | advil, aspirin, bengay, joints, prednisone |
| 52. Insomnia [Sleep/Wake] | get up every hour, keeps you awake, lyrica, stay awake, trouble falling asleep, waking up over and over |
| 53. Fatigue [Sleep/Wake] | can't get awake, dex, dexamethasone, don’t have the energy, energy back up, energy levels, finally got her awake, go back to bed, knocked her out, knocked him out, knocked me out, knocked you out, lyrica, no energy, prednisone, slept all the time, stayed in bed, steroid, steroids, take a little rest, take naps, wiped out, your energy |
| 54. Anxious [Mood] | diazepam, emotion stress, lyrica, mind is racing, nervous, no stress, nosediving, scared, valium |
| 55. Discouraged [Mood] | feel blue, nosediving, tore her up, tore him up, tore me up |
| 56. Sad [Mood] | feel blue, little bit down, lyrica, nosediving, tore her up, tore him up, tore me up |
| 58. Missed Expected Menstrual Period [Genitourinary] | yarrow root |
| 61. Painful Urination [Genitourinary] | issues with urination, peeing okay, urinary changes |
| 62. Urinary Urgency [Genitourinary] | issues with urination, peeing okay, urinary changes |
| 63. Urinary Frequency [Genitourinary] | urinate a lot, issues with urination, peeing okay, urinary changes |
| 65. Urinary Incontinence [Genitourinary] | dribble in your underwear, dribbling, get to the restroom on time, issues with urination, peeing okay, urinary changes |
| 76. Decreased Sweating [Miscellaneous] | yarrow root |

Exclusions: blood pressure, can ensure, can't ensure, cannot ensure, could ensure, deep breath, deep breaths, I ensure, they ensure, will ensure, won’t ensure, would ensure, you ensure

Symptom Keyword Library 1.2

| **PRO-CTCAE SYMPTOM** | **KEYWORDS / KEY PHRASES** |
| --- | --- |
| 1. Dry mouth [Oral] | biotene, biotene's, can’t get it wet, chewing gum, cracks, drier, dry mouth, dry throat, mouth dry, mouth dryness, mouth feeling dry, mouth is dry, mouth is even drier, mouth rinses, mouth seems dry, mouth stays dry, mouth stays real dry, parched, throat feeling dry, throat gets dry, throat gets real dry |
| 2. Difficulty swallowing [Oral] | choking, doesn't seem to go down, even drinking just even water is hard, food gets stuck, hard to get down, difficulty swallowing, dysphagia, dysphasia, esophagitis, hard time swallowing, hard to swallow, haven't been able to swallow, problem swallowing, problems swallowing, protonix, swallow, swallowing, trouble swallowing |
| 3. Mouth/throat sores [Oral] | mouth bright red, mouth is bright red, mouth is red, mouth or throat sores, mouth sore, mouth sores, mouth/throat sores, mucositis, sore throat, soreness in her mouth, soreness in his mouth, soreness in my mouth, soreness in the mouth, soreness in the throat, soreness in your mouth, stomatitis, throat pain |
| 4. Cracking at the corners of the mouth (cheilosis/cheilitis) [Oral] | cheilitis, cheilosis, cracking at the corners of the mouth |
| 5. Voice quality changes [Oral] | losing her voice, losing his voice, losing my voice, losing your voice, change in voice quality, changes in voice quality, hoarseness, voice, voice change, voice changes, voice changing, voice heaviness, voice is changing, voice quality changes |
| 6. Hoarseness [Oral] | a little horse, a little hoarse, hoarse, hoarse voice, hoarseness |
| 7. Taste changes [Gastrointestinal] | can't taste, craving, doesn’t really taste, don't enjoy meals, drown everything in hot sauce, food just turned me off, hard eating, how does food taste, little taste, no desire, no taste, smaller meals, tastes like cardboard, tastes like nothing, tastes metallic, don't taste, dysgeusia, gysguesia, problems with taste, taste changes |
| 8. Decreased appetite [Gastrointestinal] | anorexia, appetite, appetite change, appetite is just not really tere, appetite is just not there, appetite is low, appetite is not there, appetite is poor, appetite loss, appetite low, appetite off, appetite poor, appetite's, appetite's just not there, appetite's low, appetite's not there, appetite's pretty low, change in appetite, decreased appetite, decreased oral intake, don't have much appetite, don't have much of an appetite, don’t feel hungry, early satiety, eating good, food appealing, forcing things down, get hungry, hard eating, hardly eat, haven’t been hungry, haven't had an appetite, having a problem with food being appealing, lack of appetite, lose the desire to drink, lose the desire to eat, loss of appetite, lost her appetite, lost his appetite, lost my appetite, lost the desire to drink, lost the desire to eat, lost your appetite, low appetite, lower appetite, megace, no appetite, no desire, not eating, not hungry, poor appetite, poor PO intake, smaller meals, trouble eating, try even a bite, try to force things, unable to eat, wasn't appealing, yarrow root |
| 9. Nausea [Gastrointestinal] | queasiness, queasy, abhr, abhr gel, anti-nausea, compazine, inflammation of your stomach, kaopectate, kytril, meclizine, nausea gel, zofran, nausea, nauseated, nauseation, nauseous, prednisone |
| 10. Vomiting [Gastrointestinal] | abhr, abhr gel, aciphex, compazine, dry heaves, dry heaving, emesis, esomeprazole, inflammation of your stomach, keeping it down, kytril, meclizine, prednisone, puke, puked, pukes, puking, rolaids, sucralfate, threw up, throw up, throwing up, thrown up, throws up, vomit, vomited, vomiting, vomits, zofran |
| 11. Heartburn [Gastrointestinal] | acid, acid blockers, acid buildup, acid reflux, antacids, apple cider vinegar in water, burning, burning after eating, burning around the throat, dyspepsia, elevate the head, gaviscon, gaviscons, gerd, head of your bed up, heartburn, indigestion, kaopectate, magnesium, milk of magnesia, milk of magnesium, nexium, pepcid, protonix, rabeprazole, rabon, reflux, regurgitate, regurgitates, regurgitating, severe burning, stomach's talking to me, tagamet, wedge pillow, wedge pillows, zantac |
| 12. Gas [Gastrointestinal] | burp, burping, burps, pass gas, pass the gas, simethicone, flatulence, flatus, gas, gassy |
| 13. Bloating [Gastrointestinal] | bloating-ness, blows up like a balloon, bomb went off, filled up with air, how big my stomach was, inflammation of your stomach, protruded, sounds like a drum, sounds like a tom tom or a drum, stomach blows up, stomach got really big, stomach was all big, stomach was extended, stomach was really extended, stomach's all big, abdominal distension, abdominal distention, bloated, bloating, distended, distension, distention, fullness, stomach swelling, swollen abdomen |
| 14. Hiccups [Gastrointestinal] | hiccup, hiccups |
| 15. Constipation [Gastrointestinal] | activia, backed up, backed-up, benefiber, bowel obstruction, bowels, bowels moving, calcium, calciums, colace, constipa, constipated, constipates, constipating, constipation, docusate, enema, fiber, gatorade prep, gets things moving, hard as a rock, hard stool, hard stools, indigestion, lack of bowel movement, lactulose, laxative, probiotics, magnesium, makes them easier to pass, makes your stool soft, makes your stools soft, metamucil, milk of magnesia, milk of magnesium, miralax, obstipation, prune juice, senna, senokot, soften things, stool softener, stool softeners, stools, stools are hard, stools are so hard, stop up the bowels, stop up your bowels, straining, to strain, trouble pooping, when you strain, you have this blockage |
| 16. Diarrhea [Gastrointestinal] | bowels, diarrhea, diarrhea's, diarrheas, gatorade, get my bowels slowed back down, get my bowels slowed down, get your bowels slowed back down, get your bowels slowed down, imodium, imodiums, it was water, it's all watery, it's watery, kaopectate, lomotil, ned, on the loose side, pedialyte, probiotics, rumbling in the stomach, slow your bowels down, stomach is very rumbly, stool's too loose, stool's watery, stools, stools become too loose, the depends, the runs, the trots, water mixed with loose stool, watery rush, watery stool, yarrow root |
| 17. Abdominal Pain [Gastrointestinal] | a stitch, abd cramps, abd discomfort, abd pain, abdomen, abdominal cramp, abdominal cramps, abdominal discomfort, abdominal pain, bottom falls out, discomfort in her abdomen, discomfort in his abdomen, discomfort in my abdomen, discomfort in your abdomen, epigastric discomfort, epigastric pain, heating pad, left upper quadrant discomfort, left upper quadrant pain, LUQ discomfort, LUQ pain, pain in her stomach, pain in his stomach, pain in my stomach, pain in the stomach, pain in your stomach, right upper quadrant discomfort, right upper quadrant pain, RUQ discomfort, RUQ pain, stomach ache, stomach hurting, stomach hurts, stomach's hurting, stomach pains, stomachache, upset my stomach, upset stomach, upset your stomach, "when I get these pains, I know I got to shit" |
| 18. Fecal Incontinence [Gastrointestinal] | bathroom, bottom falls out, bowel, bowels, bowels moving, can't poop, comes out without even knowing it, continence, diaper, diaper pad, digest, digestive, dribble in your underwear, dribbling, fecal incontinence, get to the restroom on time, had an accident, had some accidents, have an accident, having any accidents, incontinence, incontinent, not having any continence, not having any kind of continence, panty-type things, several accidents, soiled it, specialized panties, stool, stool incontinence, stool's, stools, toilet, "when he has to go, he has to go", "when he has to go, literally he has to go", "when I have to go, I have to go", "when she has to go, she has to go" |
| 19. Shortness of Breath [Respiratory] | ability to breathe, breath, breath is deteriorating, breathe, breathe lightly, breathing, breathless, breaths, can't catch my breath, can't catch your breath, catching my breath, catching your breath, choking, DOE, dyspnea, dyspnea on exertion, dyspnea with exertion, dyspneic, exertion, exertional dyspnea, exertional shortness of breath, huffing, huffing and puffing, labored breathing, limiting my breathing, limiting your breathing, not have enough oxygen, out of breath, outta breath, oxygen, puffing, pulmicort, run short of oxygen, short of breath, short of breath with exertional activity, short of breaths, shortness of breath, shortness of breath on exertion, shortness of breath with activity, shortness of breath with exertion, SOB, SOB with exertion, take my breath away, winded |
| 20. Cough [Respiratory] | [coughing], [coughs], cough, cough suppressant, cough syrup, coughed, coughing, coughing], coughs, delsym, junky in my chest, pertussis, protonix, robitussin, strangle, strangling, strangulating, vicks |
| 21. Wheezing [Respiratory] | chest makes noise, chest making noise, heard a whistling, junky in my chest, puffing, pulmicort, wheezing |
| 22. Swelling [Cardio/Circulatory] | anti-swelling, bursitis, celebrex, dex, dexamethasone, edema, feel like I'm walking on big, fluid collection, hydrocortisone, lymphedema, prednisone, puffing, puffing up, puffy, retaining a lot of fluid, shoes got a little tight, steroid, steroids, swell, swelled, swelling, swellness, swollen, toes are squished, toradol |
| 23. Heart Palpitations [Cardio/Circulatory] | heart is starting to race, heart palpitation, heart palpitations, irregular heartbeat, irregular heartbeats, lidocaine, palpitations, racing heart, rapid heart rate |
| 24. Rash [Cutaneous] | bunch of little bumps, blotches, cellulitis, dermatitis, eruption, face rash, hydrocortisone, lesions, marks, metrocream, rash, rashes, reaction, red, red blotches, shingles, skin rash |
| 25. Skin Dryness [Cutaneous] | alligator skin, aquaphor, aveeno, breakdown of your skin, dry, dry around the heels, dry skin, dryness, eucerin, it's dry, keep it moisturized, leathery, lotion, lubiderm, lubriderm, moisturizer, peeling, scaley, skin cracking, skin dryness, skin is dry, skin is more thin, skin is so dry, skin is so thin, skin is thin, skin thin, thin skin, thin-skin working hands, Vaseline, xerosis |
| 26. Acne [Cutaneous] | acne, metrocream |
| 27. Hair Loss [Cutaneous] | a little thinner, alopecia, eyebrows, eyelashes, fall out, falling out, fuzz, hair, hair loss, hair thinning, hair's, losing her hair, losing his hair, losing my hair, losing your hair, lost all her lair, lost all his hair, lost all my hair, lost all your hair, lost hair, lost her hair, lost his hair, lost my hair, lost your hair, mustache, thinning, wig |
| 28. Itching [Cutaneous] | benadryl, don't scratch, hydrocortisone, I scratch, irritated, irritates, irritating, irritation, itch, itches, itchiness, itching, itchy, pruritic, pruritis |
| 29. Hives [Cutaneous] | blisters, bumps, hives, metrocream, urticaria |
| 30. Hand-foot Syndrome [Cutaneous] | hand-foot, hand-foot syndrome, hand/foot, hand/foot syndrome, PPE |
| 31. Nail Loss [Cutaneous] | brittle nails, fingernails, nail loss |
| 32. Nail Ridging [Cutaneous] | fingernails, like rings, nail ridging, ridges, the rings |
| 33. Nail Discoloration [Cutaneous] | become darker sometimes, fingernails, gonna be darker, like rings, nail changes, nail discoloration, the darkness, the rings |
| 34. Sensitivity to Sunlight [Cutaneous] | sensitivity to sunlight, eucerin, sunburn |
| 35. Bed/pressure Sores [Cutaneous] | bed sore, bed sores, bedsore, bedsores, blisters, pressure sore, pressure sores, sore, sores |
| 36. Radiation Skin Reaction [Cutaneous] | dry skin comes up shaped just like the incision, injury from radiation, radiation injury, radiation skin reaction, radiation skin reactions, radiation treatment affected tissue, radiation-injured, skin toxicity |
| 37. Skin Darkening [Cutaneous] | become darker sometimes, cortisone, color change, erythema, flushing, funniest looking spot, gonna be darker, hyperpigmentation, hyperpigmented, jaundiced, redness, rosy cheeks, skin darkening, skin darker, spot on your skin, the darkness |
| 38. Stretch Marks [Cutaneous] | stretch marks |
| 39. Numbness & Tingling [Neurological] | 10 mg PO, are more numb, are number, bengay, burning, burning pain, carpal tunnel, compression damage, compression sleeve, compression sock, compression stocking, cymbalta, didn’t feel it, dysesthesia, dysesthesias, dysesthia, feet and legs, fingertips, gauntlet, in the fingers, in the sole of the feet, just like buzz, less sensation, lidocaine, longer gauntlet, lose feeling, lyrica, needles and pins, nerve damage, nerve injury, nerve pain, nerves are doing better, neuralgia, neuropathic, neuropathic pain, neuropathy, nucynta, numb, numbness, numbness & tingling, numbness and tingling, numbness or tingling, numbness/tingling, paresthesia, paresthesias, permanent sensation, pins and needles, PN, recover my feelings, sciatica, stocking glove, tingling, tingling and numbness, tingly, tips of my fingers, tips of your fingers |
| 40. Dizziness [Neurological] | black out, blackout, collapsed, collapsing, dizziness, dizzy, faint, fainted, fainting, knocked her out, knocked him out, knocked me out, knocked you out, meclizine, light headed, light-headed, lightheaded, lightheadedness, lose consciousness, loss of consciousness, pass out, passed out, passing out, room's spinning, spell where you collapsed, vertigo, wobbly, woozy |
| 41. Blurred Vision [Visual/Perceptual] | blurred, blurred vision, blurry vision, cataract, diplopia, double-vision, keep seeing stuff, problem with her vision, problem with his vision, problem with my vision, problem with vision, problem with your vision, vision, vision gray out, vision grays out, visual disturbance, visual disturbances |
| 42. Flashing Lights [Visual/Perceptual] | flashing lights, lights flashing, see funny things, see lights, see lights or funny things |
| 43. Visual Floaters [Visual/Perceptual] | floaters, visual floaters |
| 44. Watery Eyes [Visual/Perceptual] | eyes are watery, eyes water, eyes watering, eyes watery, tear up, watering eyes, watery eyes |
| 45. Ringing in Ears [Visual/Perceptual] | ears ringing, ringing in ears, ringing in her ears, ringing in his ears, ringing in my ears, ringing in your ears, tinnitus |
| 46. Concentration [Attention/Memory] | ability to focus, alert, alertness, attention, chemo brain, cognitive, cognitive slowing, cognitively, cognitively slower, concentrate,concentration, confused, confusion, decreased concentration, difficulty concentrating, difficulty focusing, disoriented, distracted, focused and paying attention, fog, fuzzy brain, goofiness, hard to concentrate, loopy, mental plasticity, mental stamina, mental thing, mental things, mixing up, more alert, out of it, paying attention, problems with understanding, problems with your memory or your thinking, process of thinking, trouble concentrating, trouble really concentrating, trouble with thinking |
| 47. Memory [Attention/Memory] | difficulty remembering, forgetful, forgetfulness, memory, memory loss, memory's, mushkabobble, problems with your memory or your thinking, short-term memories, short-term memory |
| 48. General Pain [Pain] | acetaminophen, ache, aches, aches/pains, achiness, aching, achy, advil, aggravating, agitates, agitating, agitation, aleve, aspirin, balled up, bengay, breakthrough, bone grinding, bothering me, buckling over, celebrex, chest discomfort, chest heaviness, chest pain, chest pain and heaviness, chest pressure, choking, codeine, codeine's, codone, cortisone, CP, cutting, cymbalta, darvocet, dex, dexamethasone, dilaudid, discomfort, drives me bananas, epidurals, excruciating, feel really bad, fentanyl, generalized pain, gnawing, hurt, hurting, hurts, hydrocodone, hydromorphone, ibuprofen, ibuprofen/motrin, it burns, kind of shoot, LBP, like arrows, methadone, morphine, motrin, naproxen, narcotic, narcotics, norco, oxy, oxy's, oxycodone, oxycodones, oxycontin, pain, pain-medicine, pain's, painful, pains, percocet, pressure, pressure's, radiating up, sensitivity, sharp, sharpness, shooting, sore, soreness, spiking, stabbing, steroid, steroids, tender, tenderness, toradol, tramadol, twinge, twinges, tylenol, uncomfortable, vicodin |
| 49. Headache [Pain] | advil, aspirin, excedrin, HA, head feels heavy, headache, headaches, heavy head, migraine, migraines |
| 50. Muscle Pain [Pain] | a stitch, ache, aches, achiness, bengay, body aches, charley horse, charley horses, cramp, cramping, cramps, crampy, cymbalta, diazepam, muscle ache, muscle aches, muscles are sore, muscle pain, muscle spasm, muscle spasms, muscle tension, magnesium, myalgia, myalgias, naproxen, quinine, spasm, spasms, tonic, twinge, twinges, valium, vicks |
| 51. Joint Pain [Pain] | advil, aleve, arthralgia, arthralgias, arthritic pain, aspirin, bengay, bursitis, celebrex, corticosteroid, cortisone, ibuprofen, ibuprofen/motrin, in the joint, joint discomfort, joint issue, joint issues, joint pain, joint pains, joints, joints are hurting, joints hurt, knee pain, motrin, naproxen, painful joints, prednisone, tendinitis, tendonitis |
| 52. Insomnia [Sleep/Wake] | ambien, ativan, benadryl, can't sleep, cannot sleep, cat napping, couldn't sleep, didn't sleep very well, difficulty falling asleep, difficulty sleeping, difficulty staying asleep, difficulty with sleep, disrupted sleep, don't get sleepy, don't get tired, dyssomnia, falling asleep issue, get up every hour, insomnia, interruptions in her sleep, interruptions in his sleep, interruptions in my sleep, interruptions in your sleep, keep me awake, keeping her awake, keeping him awake, keeping me awake, keeping you awake, keeps me awake, keeps you awake, lorazepam, lyrica, melatonin, not been sleeping, not really sleeping, not sleeping, poor sleep, sleep, sleep deprivation, sleep disturbance, sleep disturbances, sleeping, sleeping pill, stay awake, still awake at, tossing and turning, trouble falling asleep, trouble sleeping, up all night, wake up in the middle of the night, waking up in the middle of the night, waking up over and over |
| 53. Fatigue [Sleep/Wake] | all the juice is gone, can’t get awake, decreased energy, dex, dexamethasone, doesn't have much stamina, don't have much stamina, don't have stamina, don’t have the energy, dopey, dozing off, draggy, drowsy, energy back up, energy level, energy levels, exertion, exhausted, fatigue, fatigued, finally got her awake, go back to bed, groggy, has to rest more often, have to rest more often, help with staying awake, helps with staying awake, I don't last very long, knock me out, knock you out, knocked her out, knocked him out, knocked me out, knocked you out, knocks her out, knocks him out, knocks me out, knocks you out, lack of energy, less energies, less energy, less stamina, low energy, lyrica, make her sleepy, make him sleepy, make me sleepy, make you sleepy, makes her sleepy, makes him sleepy, makes me sleepy, makes you sleepy, more sleepy, no energy, passing out, poop out, prednisone, run out of juice, sedation's really high, sleep all day, sleepiness, sleeps all day, sleepy, slept all the time, stayed in bed, steroid, steroids, take a little rest, take naps, tire, tire more easily, tired, tiredness, tiring, too tired, unconscious, wiped out, wipes her out, wipes him out, wipes me out, wipes you out, wiping her out, wiping him out, wiping me out, wiping you out, worn down, your energy, zapped |
| 54. Anxious [Mood] | afraid, agitation, anxieties, anxiety, anxiety symptoms, anxious, ativan, buspirone, can't relax, cymbalta, diazepam, emotion stress, get panicked, gets panicked, jumpy, lorazepam, lyrica, make you relax, mind is racing, mirtazapine, nervous, nervousness, no stress, nosediving, panic, panic attack, panic attacks, panic disorder, panic spell, restless, scared, start shaking inside, valium, worried, worries, worry, worrying, xanax, zoloft |
| 55. Discouraged [Mood] | discouraged, disheartening, feel blue, hopeless, hopelessness, listless, mood disorder, mood disorders, nosediving, overwhelmed, tore her up, tore him up, tore me up |
| 56. Sad [Mood] | amitriptyline, anguish, anti-depressant, anti-depressants, antidepressant, antidepressants, celexa, citalopram, cries, crying, cymbalta, depressed, depressed mood, depressing, depression, depressive, depressive sxs, depressive symptoms, down in the dumps, dysphoria, dysphoric mood, feel blue, feel down, feeling a little more down, feeling blue, feeling down, feeling kind of blue, feeling kind of low, feeling low, grief, grieving, hurting yourself, little bit down, locked up in the crazy house, low mood, low/depressed, low/depressed mood, lyrica, mental facility, mirtazapine, mood, mood disorder, mood disorders, mood has been low, mood instability, mood is down, mood is low, mood issues, mood symptoms, mood toxicity, mood's been down, negative frame of mind, nosediving, not herself mentally, not himself mentally, not myself mentally, not yourself mentally, psychiatric evaluation, psychiatric problems, psychologic, remeron, sad, sadness, seroquel, suicidal, suicidal thoughts, tearful, tearfulness, teary, tore her up, tore him up, tore me up, zoloft |
| 57. Irregular Periods/Vaginal Bleeding [Genitourinary] | bleeding, irregular period, irregular periods, postmenopausal bleeding, vaginal bleeding, vaginal spotting |
| 58. Missed Expected Menstrual Period [Genitourinary] | menopause, menses, missed expected menstrual period, missed expected period, missed her period, missed my period, missed period, missed your period, yarrow root |
| 59. Vaginal Discharge [Genitourinary] | bloody discharge, some discharge, vaginal discharge |
| 60. Vaginal Dryness [Genitourinary] | vaginal dryness |
| 61. Painful Urination [Genitourinary] | hurt to pee, hurts to pee, issues with urination, pain during urination, pain while urinating, painful urination, peeing okay, urinary changes |
| 62. Urinary Urgency [Genitourinary] | changes in your urinary habits, issues with urination, peeing okay, urinary changes, urinary urgency |
| 63. Urinary Frequency [Genitourinary] | bladder, changes in your urinary habits, emptying her bladder, emptying his bladder, emptying my bladder, emptying your bladder, frequent urination, issues with urination, loss of bladder control, nocturia, peeing okay, polyuria, urinary changes, urinary frequency, urinate a lot, urinating more frequently |
| 64. Change in Usual Urine Color [Genitourinary] | change in usual urine color, change in your urine, changes in your urine, unusual urine color |
| 65. Urinary Incontinence [Genitourinary] | bathroom, catheter, catheterization, changes in your urinary habits, comes out without even knowing it, dribble in your underwear, dribbling, every time I cough, get to the restroom on time, had an accident, had some accidents, have an accident, having any accidents, I feel like I'm peeing, incontinence, incontinent, issues with urination, leaking, not having any continence, not having any kind of continence, panty-type things, peeing all over the place, peeing okay, several accidents, specialized panties, trouble urinating, urinary changes, urinary incontinence, uropathy, "when he has to go, he has to go", "when he has to go, literally he has to go", "when I have to go, I have to go", "when she has to go, she has to go" |
| 66. Achieve and Maintain Erection [Sexual] | achieve and maintain erection, erectile dysfunction |
| 67. Ejaculation [Sexual] | ejaculation |
| 68. Decreased Libido [Sexual] | decreased libido, low libido, no sex drive |
| 69. Delayed Orgasm [Sexual] | delayed orgasm |
| 70. Unable to have Orgasm [Sexual] | unable to have orgasm |
| 71. Pain w/ Sexual Intercourse [Sexual] | dyspareunia, pain during intercourse, pain during sex, pain during sexual intercourse, pain with sexual intercourse |
| 72. Breast Swelling and Tenderness [Miscellaneous] | breast pain, breast swelling, breast swelling and tenderness, breast swelling or tenderness, breast tenderness, discomfort, discomfort at the lumpectomy bed, swelling, swollen |
| 73. Bruising [Miscellaneous] | black and blue, black-and-blue, blue-ish, bruise, bruises, bruising |
| 74. Chills [Miscellaneous] | arctic, can't get warm, cannot get warm, chill, chilled, chilling, chills, cold and chilled, cold sensation, couldn't get warm, feel cold all the time, freezing, hot and cold, hot cold, hot/cold, shakiness, shaking, shiver, shivering, shivering/shaking, takes a while to get warm, teeth are chattering, teeth chattering, teeth have been chattering |
| 75. Increased Sweating [Miscellaneous] | codeine, diaphoresis, diaphoretic, increased sweating, night sweats, pillow is all wet, pillow is soaked, pillow is wet, soaked, soaking, soaking wet, sweat, sweating, sweats |
| 76. Decreased Sweating [Miscellaneous] | decreased sweating, less sweating, yarrow root |
| 77. Hot Flashes [Miscellaneous] | hot flash, hot flashes, hotflashes, temperature deregulation, temperature dysregulation, warming of the body |
| 78. Nosebleed [Miscellaneous] | bloody nose, bloody noses, epistaxis, nosebleed, nosebleeds |
| 79. Pain and Swelling at Injection Site [Miscellaneous] | injected, injection site, pain and swelling at injection site |
| 80. Body Odor [Miscellaneous] | body odor |

Exclusions: blood pressure, can ensure, can't ensure, cannot ensure, could ensure, deep breath, deep breaths, don’t worry, I ensure, red blood cell(s), they ensure, will ensure, would ensure, you ensure

Symptom Keyword Library 1.2.0 – Clinician-Authored Language to Review and Remove from Library if Desired

| 10 mg PO, abd cramps, abd discomfort, abd pain, abdominal cramps, abdominal discomfort, abdominal distension, abdominal distention, aches/pains, alopecia, anorexia, anxieties, appetite change, appetite loss, appetite low, appetite poor, arthralgia, arthralgias, arthri, arthritic pain, bedsore, bleeding, bloating, bloody noses, blurry vision, body aches, breast pain, brittle nails, burning pain, cellulitis, change in appetite, chest discomfort, chest pain, chest pain and heaviness, chest pressure, color change, CP, cries, decreased concentration, decreased energy, decreased oral intake, depressive, depressive sxs, depressive symptoms, dermatitis, diaphoresis, diaphoretic, difficulty concentrating, difficulty falling asleep, difficulty remembering, difficulty staying asleep, diplopia, discomfort at the lumpectomy bed, discomfort in his abdomen, discouraged, disrupted sleep, distension, distracted, DOE, dry heaves, dry heaving, dysesthesia, dysesthesias, dysesthia, dysgeusia, dyspareunia, dyspepsia, dysphasia, dysphoria, dysphoric mood, dyspnea, dyspnea on exertion, dyspnea with exertion, dyspneic, dyssomnia, early satiety, edema, emesis, epigastric discomfort, epigastric pain, epistaxis, erectile dysfunction, erythema, esophagitis, exertional dyspnea, exertional shortness of breath, fecal incontinence, feeling low, flatulence, flatus, floaters, flushing, food gets stuck, forgetful, forgetfulness, frequent urination, fullness, generalized pain, gout, grief, grieving, gysguesia, HA, hair loss, hair thinning, hand-foot syndrome, heart palpitation, hiccup, hiccups, hoarse, hoarse voice, hopeless, hopelessness, hot flash, hotflashes, hurting, hyperpigmentation, hyperpigmented, increased swelling, irregular heartbeats, itchiness, joint discomfort, joint pains, LBP, left upper quadrant discomfort, left upper quadrant pain, light headed, low appetite, low energy, low libido, low mood, low/depressed, low/depressed mood, lower appetite, LUQ discomfort, LUQ pain, lymphedema, memory loss, mood has been low, mood is low, mood symptoms, mouth sore, mouth sores, mucositis, muscle spasm, muscle spasms, muscle tension, myalgia, myalgias, nail changes, ned, nerve pain, neuralgia, neuropathic, neuropathic pain, no appetite, no sex drive, nocturia, nosebleeds, not been sleeping, not eating, numbness & tingling, numbness/tingling, obstipation, painful joints, paresthesia, paresthesias, peeling, pertussis, PN, polyuria, poor PO intake, poor sleep, postmenopausal bleeding, pruritic, pruritis, queasiness, racing heart, right upper quadrant discomfort, right upper quadrant pain, RUQ discomfort, RUQ pain, sadness, sciatica, shivering/shaking, short of breath with exertional activity, short of breaths, shortness of breath on exertion, shortness of breath with activity, shortness of breath with exertion, skin cracking, skin dryness, sleep deprivation, sleep disturbance, sleep disturbances, SOB, SOB with exertion, spasm, spasms, stool incontinence, tearful, tearfulness, teary, throat pain, tinnitus, trouble eating, trouble sleeping, trouble swallowing, unable to eat, urinary frequency, urinary incontinence, urinary urgency, urticaria, vaginal bleeding, vaginal discharge, vaginal dryness, vaginal spotting, vertigo, voice change, voice changes, voice heaviness, vomited, vomits, watery eyes, xerosis |
| --- |
