# Supplementary material for: Development of a keyword library for capturing PRO-CTCAE-focused “symptom talk” in oncology conversations
Source: JAMIA Open. 2023 Feb 9;6(1):ooad009. doi: 10.1093/jamiaopen/ooad009 (PMC9912707; doi:10.1093/jamiaopen/ooad009)
Supplement: ooad009_Supplementary_Data [file ooad009_supplementary_data.zip › SKL_Supplemental_Table1_11_02_22.docx]

**Supplemental Table 1:** Codebook for Qualitative Content Analysis of Misclassified Turns and True Positives

| ***Sample*** | ***Category*** | ***Sub-category*** | ***Examples relevant to PRO-CTCAE symptoms*** | ***Examples not or ambiguously relevant*** |
| --- | --- | --- | --- | --- |
| **False Negatives** | Not a discrete symptom | General symptom discussion (including absence of symptom) | N/A | “doing well”, “gets better”, “feel better”, “how are you feeling today”, “anything new been bugging you” |
|  |  | Description of symptom/quality | “things will just run through” (diarrhea), “churning up” (nausea), “gassed” (fatigue) | “gaining weight”, “losing weight”, “points”, “the other agony”, “odor”, “yellow-ish stuff”, “miserable” |
|  |  | Description of symptom/severity | N/A | “couldn’t get up”, “in bed for a month”, “so weak”, “saw a psychologist”, “kill you” |
|  |  | Description of symptom/location | “hands”, “feet”, “in my hand”, “your toes” (likely references to neuropathy) | “armpit”, “top of my back”, “head has healed” |
|  |  | Description of symptom/frequency | N/A | “at night” |
|  |  | Description of symptom/duration | N/A | “wasn’t healing” |
|  | Symptom management | Non-pharm and pharm strategies | “compression sleeve”, “Neurontin”, “simethicone”, “valium” | “antiseptic”, “hot shower”, “lying still”, “lying flat” |
|  | Discrete symptom | Actual symptom words | “toenails turn colors”, “swells”, “bowels are doing okay”, “accidents”, “weakness” (neuro) | “myasthenia”, “bigger and bigger lump there”, “fever” |
|  | Symptom response | Replies to a previously relevant symptom turn | “seven and three quarters”, “uh, well I would say a nine” (pain ratings) | “yes”, “no”, “mess up”, “most”, “right”, “yeah”, “a little” |
|  | Coder (human annotator) error | Conversational symptom discussion (i.e., continuers that don't contain symptom-relevant words) | N/A | “I know a colonoscopy is on your horizon”, “clot”, “shoot?” |
|  |  | Medications that aren’t symptom-relevant | N/A | “lantus”, “novolog”, “arixtra”, “antibiotics”, “vitamin D3” |
|  |  | Signs vs. symptoms | N/A | “low hemoglobin”, “thrush”, “osteonecrosis”, “dehydration”, “hypertension”, “diabetes” |
|  |  | Other | N/A | “I don’t know how I am” |
| **False Positives** | Coder (human annotator) error | Medication miss (pharm or non-pharm symptom management) | “long-acting narcotic”, “narcotics”, “nexium”, “oxycodone”, “stool softeners”, “make sure you get enough sleep at night” | N/A |
|  |  | Symptom assessment | “bowels”, “straining”, “pushing your pee”, “bleeding” | N/A |
|  |  | Symptom description | “sore”, “painful” | N/A |
|  |  | Discrete symptom miss | “bowel”, “good mood”, “don’t have the appetite”, “poop out”, “so tired”, “pain is not getting any better”, “pain” | N/A |
|  | Physical exam | Symptom-containing words during the physical exam | “deep breaths”, “breathe”, “pain when I press”, “lots of pressure”, “uncomfortable”, “bleeding” | |
|  |  | Transcribed cough | “cough”, “[cough]” | N/A |
|  | Symptom-containing turn, out of context | Symptom descriptor | N/A | “pressure”, “your pressure”, “blood pressure”, “shooting people”, “red one”, “red” |
|  |  | Conversational use of symptom terms | N/A | “bothering you”, “nervous”, “worry/worried”, “reaction”, “don’t worry”, “don’t get discouraged”, “chills”, “out of it”, “hurt anything”, “hair”, “memory” |
|  |  | Symptom location (ambiguous) | N/A | “abdomen”, “bowel”, “bladder”, “cough” |
|  | Library error | Ambiguous terms that should be excluded from the library | N/A | “calcium”, “magnesium”, “Gatorade”, “probiotics”, “injected” |
|  |  | Signs vs. symptoms | N/A | “cataracts”, “dementia” |
| **True Positives** | PRO-CTCAE symptom description or medication | Oral (dry mouth; difficulty swallowing; mouth/throat sores; cracking at the corners of the mouth; voice quality changes; hoarseness) | N/A | N/A |
|  |  | GI (taste changes, decreased appetite, nausea, vomiting, heartburn, gas, bloating, hiccups, constipation, diarrhea, abdominal pain, fecal incontinence) | “stool softener”, “nausea”, “explosion of gas”, “loose bowel movements”, nausea medicine”, “stomachache” | “will increase your appetite”, “changes to the bowel”, “moving your bowels okay” |
|  |  | Respiratory (shortness of breath; cough; wheezing) | “short of breath”, “problems with cough”, “trouble breathing” | N/A |
|  |  | Cardio / Circulatory (swelling; heart palpitations) | “swelling”, “swollen” | N/A |
|  |  | Cutaneous (rash; skin dryness; acne; hair loss; itching; hives; hand-foot syndrome; nail loss; nail ridging; nail discoloration; sensitivity to sunlight; bed/pressure sores; radiation skin reaction; skin darkening; stretch marks) | “alopecia”, “hair fell out”, “itching”, “skin changes”, “redness”, “shiny skin on your hands or feet” | “sores” |
|  |  | Neurological (numbness & tingling; dizziness) | “neuropathy” | “feet was hurting so bad”, “it’s numb”, “coming from my nerves” |
|  |  | Visual / Perceptual (blurred vision; flashing lights; visual floaters; watery eyes; ringing in ears) | “did you have blurred vision” | N/A |
|  |  | Attention / Memory (concentration; memory) | N/A | “okay. Concentrate.” |
|  |  | Pain (general pain; headache; muscle pain; joint pain) | “oxycodone”, “oxycontin”, “Tylenol”, “any pain”, “hurting so bad”, “began to hurt”, “hurting”, “muscles hurt”, “cramping”, “pain pill”, “spasms in my back” | N/A |
|  |  | Sleep / Wake (insomnia; fatigue) | “can’t sleep”, “more tired than normal” | “want to sleep”, “little bit tired” |
|  |  | Mood (anxious; discouraged; sad) | “dealing with anxiety and depression”, “not been depressed. Not been upset.”, “you doing okay, mentally? Do you need something for anxiety?”, “high anxiety” | “worry”, “worried” |
|  |  | Genitourinary (irregular periods/vaginal bleeding; missed expected menstrual period; vaginal discharge; vaginal dryness; painful urination; urinary urgency; urinary frequency; change in usual urine color; urinary incontinence) | “vaginal bleeding, discharge”, “urgency for the bathroom” | “peeing a lot” |
|  |  | Sexual (achieve and maintain erection; ejaculation; decreased libido; delayed orgasm; unable to have orgasm; pain w/ sexual intercourse) | N/A | N/A |
|  |  | Miscellaneous (breast swelling and tenderness; bruising; chills; increased sweating; decreased sweating; hot flashes; nosebleed; pain and swelling at injection site; body odor) | “how about flushing, sweating”, “night sweats”, “hot flashes” | N/A |
|  | General symptom/ medication mention | | “prednisone”, “steroid gives me a hard time”, “decadron”, “dry” | |
|  | Non-PRO-CTCAE symptom description or medication | | N/A | “blood pressure” |
|  | Library error | Symptom-containing turn, out of context | N/A | “it’s aching ya?” |
|  |  | Physical exam | “does it hurt when I press”, “a lot of pressure. You had any pain?” | |
|  |  | Word to be removed from library (signs vs. symptoms, ambiguous medication issues) | N/A | “gout”, “arthritis”, “calcium” |
