# Supplementary material for: Development of a keyword library for capturing PRO-CTCAE-focused “symptom talk” in oncology conversations
Source: JAMIA Open. 2023 Feb 9;6(1):ooad009. doi: 10.1093/jamiaopen/ooad009 (PMC9912707; doi:10.1093/jamiaopen/ooad009)
Supplement: ooad009_Supplementary_Data [file ooad009_supplementary_data.zip › SKL_Appendix_A_11_02_22.docx]

**APPENDIX A: Supplemental Methods**

**Symptom Domain Codebook for Human Annotation**

| *Definition* | “Symptom talk” includes all references to symptoms and side effects of both cancer and its treatment, whether potential or actual (e.g., appetite, bleeding, cachexia/weight loss, shortness of breath/cough, edema emotional and mood symptoms, fatigue, general symptoms, GI/GU, nausea/vomiting, neurological symptoms (e.g. weakness/memory problems), pain, sexuality/intimacy, sleep, hair loss; allergy symptoms; skin symptoms (e.g., skin discoloration)). Symptom talk also includes negation of symptoms and discussion of symptoms generally, even if they are not related to the patient (e.g., a friend's symptoms) or not related to serious illness.  Symptom talk can include a global assessment of symptoms.  Symptom talk includes statements related to *symptom management:* annotators should include turns mentioning medications if the medication is named, or if the symptom is named in reference to the medication. Annotators should not include logistics of prescriptions or dosing unless the medication is named.  Symptom talk includes statements related to *future symptoms* (e.g. anticipatory guidance about disease trajectory or treatment side effects, and management of future symptoms as well).  Within symptom talk, symptom status can be experienced, not experienced, of unknown experience, or of theoretical experience. |
| --- | --- |
| *Illustrative Examples* | *Patient: I have pain in my belly*  *Clinician: So how have you been feeling since we met last?*  *Clinician: You’re not needing any pain medications?*  *Clinician: And with Revlimid, the main side effects tend to be fatigue and GI upsets. So constipation or diarrhea can happen. You may also encounter some muscle and joint problems on it, something like the bursitis but it’ll be more diffused than just localized, mostly the legs. So, again, those things, you can try Motrin or Aleve or Tylenol, if necessary. Massage helps, hot bath or hot shower helps.* |
| *Counter-Examples* | Annotators should not include discussion of “side effects” without specifics mentioned, e.g.  *Clinician: That would be an option, but that’s also not curative and really –*  *and it also has a lot of side effects that we’d be exposing you to.* |
| *Additional Notes* | Definition of symptom: subjective evidence of disease or physical disturbance observed by the patient; a departure from a patient’s normal functioning or feeling. Broadly: a symptom is something that indicates the presence of a physical disorder.^44^ |
