# Supplementary figures and images for: Development of a keyword library for capturing PRO-CTCAE-focused “symptom talk” in oncology conversations
Source: JAMIA Open. 2023 Feb 9;6(1):ooad009. doi: 10.1093/jamiaopen/ooad009 (PMC9912707; doi:10.1093/jamiaopen/ooad009)

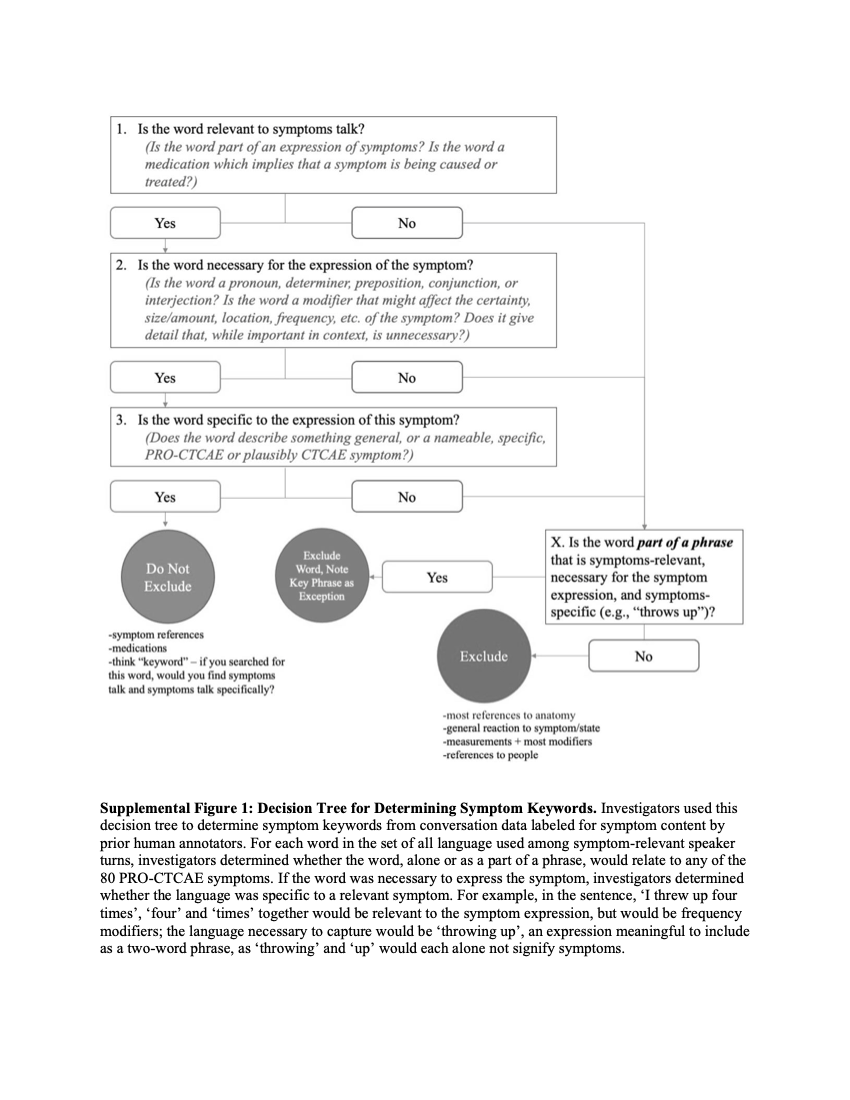

Supplement: ooad009_Supplementary_Data [file ooad009_supplementary_data.zip › SKL_Supplemental_Figure_1_11_02_22.tiff]
